# MMBRG Protocol – Infants and Preschoolers: Myofunctional Orofacial Clinic Examination

**DOI:** 10.1590/2317-1782/20212020325

**Published:** 2022-06-17

**Authors:** Andréa Monteiro Correia Medeiros, Irene Queiroz Marchesan, Katia Flores Genaro, Íkaro Daniel de Carvalho Barreto, Giédre Berretin-Felix

**Affiliations:** 1 Departamento de Fonoaudiologia, Universidade Federal de Sergipe – UFS - São Cristóvão (SE), Brasil.; 2 CEFAC Saúde e Educação – São Paulo (SP), Brasil.; 3 Departamento de Fonoaudiologia – Faculdade de Odontologia de Bauru – FOB, Universidade de São Paulo – USP - Bauru (SP), Brasil; 4 Programa de Pós-graduação em Biometria e Estatística Aplicada, Universidade Federal Rural de Pernambuco – UFRPE - Recife (PE), Brasil.

**Keywords:** Speech, Language and Hearing Sciences, Infant, Child, Preschool, Validation Studies, Myofunctional Therapy, Stomatognathic System

## Abstract

**Purpose:**

To present the Myofunctional Orofacial Clinical Examination Protocol belonging to the MMBGR Protocol - Infants and Preschoolers, including its validation.

**Methods:**

Initially, test content-based validity was evaluated from the MBGR Protocol to be used with the age group between 6 and 71 months based on the bibliography and experience between the authors (original and current). For the content and appearance analysis, 10 speech therapists specialized in Orofacial Motricity attended and filled out an electronic form with dichotic and Likert scale questions in two moments. We used the Content Validity Index and the Exact Binomial Test. Then there was a validity based on the response processes analysis followed by a reliability of the Clinical Examination with 155 participants by 7 experienced and calibrated speech therapists, and the examiners between and within agreement was verified by the Intraclass Correlation Coefficient.

**Results:**

There were additions, modifications, and exclusions of items according to the age group, resulting in the Myofunctional Orofacial Clinical Examination Protocol for Infants and Preschoolers, which obtained 90.5% agreement; and 100% of the appropriate scores by at least 90% of the specialists. In reliability, most items of the Extraoral and Intraoral Examination and Chewing obtained a reasonable to good, or even excellent, agreement.

**Conclusion:**

The “Clinical Myofunctional Clinical Examination” was validated based on the test content, response process, and reliability and, along with the “Instructional” and the “Clinical History” is part of the “MMBGR Protocol - Infants and Preschoolers” for speech therapy activities in the age group between 6 and 71 months of age.

## INTRODUCTION

Clinical examination is essential in speech therapy for establishing diagnosis and prognosis in the area of Orofacial Motricity (OM). Standardized instruments for clinic and research enable the speech therapist to plan, document, and analyze the evolution and effectiveness of the therapeutic process^(1)^. Test validation is critical in accordance with established parameters^(2)^. When it comes to the Speech-Language Pathology test, it has been suggested that validation studies include the following steps: Evidence of validity based on content, internal consistency, and relationship with other variables; Validity evidence based on response processes Reliability/accuracy; Equity; Accuracy; and respective Validity evidence based on test results^(2)^.

In the area of OM in breastfeeding, instruments have been developed to monitor the mother-newborn dyad^(3)^ and assess readiness for breastfeeding in newborns, including at-risk cases^(4-6)^; in addition to specific morphophysiological aspects^(7)^. On the other hand, standardized protocols for orofacial myofunctional assessment aimed at the population from 6 years of age are already widely recognized in speech therapy such as OMES-E^(8,9)^ and the MBGR^(10,11)^.

However, no Brazilian publication containing a standardized and validated instrument in the OM area that was focused at the age group between 6 months and 5 years and 11 months of life has been found thus far, revealing a significant gap.

Given the scarcity of standardized instruments for OM in infants and preschoolers, the goal of this paper is to present the final version of the “Orofacial Myofunctional Clinical Examination,” which forms part of the “MMBGR Protocol - Infants and Preschoolers,” demonstrating test content validation, evidence of validity based on response processes, and reliability.

## METHODS

This descriptive study is part of a research project approved by the Universidade Federal de Sergipe's Ethics and Research on Human Beings Committee under CAEE No. 12529419.6.0000.5546. The Informed Consent Form (FICF) was signed by all participants and/or guardians. This is the validation of a new instrument adapted from the MBGR protocol^(11)^ for the infant and preschool population, in accordance with the guidelines of the validation studies^(2)^, after obtaining a written opinion favorable to the adaptation from the authors of the original MBGR protocol^(11)^.

There was initially a validity step based on the test content. The new instrument was organized based on a theoretical study and the researcher's experience, with review and consensus among authors (original and current versions). A search on the Scielo, Pubmed, and Bireme platforms from 1993 to 2017 yielded a review of the literature on orofacial myofunctional development and stomatognathic functions at an early age. Speech Therapy, Infants, Preschool, Methods of Evaluation, and Stomatognathic System were the keywords.

The instrument was subjected to an appearance and content analysis. This stage included ten OM-experienced specialist speech therapists. The following were considered as inclusion criteria: have more than five years of experience in Speech-Language Pathology and/or teaching activity; have degrees and/or publications in the OM area. Non-delivery of opinions within the specified deadlines serves as exclusion criterion.

The majority of them (90 percent) had more than 15 years of experience, at least 5 years of teaching experience, and experience working with infants (80 percent) and preschoolers (80 percent). These professionals are spread across four regions of Brazil (the Midwest, Northeast, Southeast, and South); 80 percent have a Doctor's degree and 20 percent have a Master's degree. The majority (70%) are between the ages of 41 and 50.

In the validation based on test content analysis, an electronic form with dichotic questions (yes/no) was used, with fields to justify the negative answers (describing the aspect that did not agree with a given item, which could suggest modification). The Content Validity Index (CVI) and the Exact Binomial Test were used, with a minimum level of agreement of 70%. A second round of instrument analysis was performed, this time using a Likert scale^(2,12)^ with five response options (strongly agree, agree, indifferent, disagree, and strongly disagree).

The validity analysis was followed by the reliability analysis of the Orofacial Myofunctional Clinical Examination, which was carried out by seven speech therapists with experience in the assessment of OM in children under the age of six, based on the analysis of standardized images.

Images of individual clinical examinations of children, lasting approximately 30 minutes, were recorded for this purpose by the researcher (evaluator 1). Inclusion criteria: the infant and/or preschooler must be healthy and have no neurological issues. Exclusion criteria include the minor's/refusal guardian's to undergo the Orofacial Myofunctional Clinical Examination in its entirety or in part.

According to the eligibility criteria and FICF signature, 260 infants and preschool children were recruited. 46 did not accept the assessment (either partially or completely), and 10 had an incompatible image record for analysis. Of the 204 evaluated subjects with compatible images, 155 infants and preschoolers were considered, 93 (60%) from Sergipe and 62 (40%) from São Paulo, divided into age groups: 6 to 11 months (N=35); 12 to 23 months (N=35); 24 to 35 months (N=35); and 36 to 71 months (N=50).

Data was collected in four institutions: two daycare centers in the city of Bauru, in the interior of the state of São Paulo; one crèche in the city of São Cristóvão, in the state of Sergipe; and the children's clinic of the University Hospital of the Universidade Federal de Sergipe in Aracaju, which provided a room for the procedure.

The sitting position on a chair, compatible with the child's height, with the child's feet on the floor, was standardized for data collection. The infant was usually placed on the caregiver's lap, with its back and head supported and its face turned toward the examiner. In some cases involving preschool children, the procedure was also carried out in the presence and/or on the lap of the teacher, nursery assistant, or person in charge. A puppet and a toy were used to create a playful environment and to entice the child to approach. However, it was ensured that all assessment procedures were followed and recorded in a consistent manner.

Two other duly trained and calibrated evaluators recorded static (JPEG) and dynamic (MP4) images with a digital camera (Panasonic Compact-VHS Palmcorder) in their hands, with an approximate image of the orofacial region (Macro Led lens Ring Flash HD). The nomination test was filmed using a tripod. Based on previous training provided by the researcher, a group of 12 students from the Health field edited these images. The researcher reviewed all records to see if they were compatible with completing the new assessment instrument.

The edited images were shared with 7 evaluator speech therapists for reliability analysis. Evaluator 1 (A1) (principal researcher), regarded as a specialist, analyzed all of the cases in the study, while the other six Evaluators (A) 2, 3, 4, 5, 6, and 7 were distributed by age group: 6 to 11 months (A2); 12 to 23 months (A3); 24 to 35 months (A4, A5, or A6); 36 to 71 months (A6 or A7), forming a pair with A1, with a second evaluator analyzing each case.

Previously, the calibration procedure was carried out between the evaluators in accordance with the guidelines for the analysis of each aspect observed, by age group. Following calibration, each pair of evaluators independently applied the protocol with the same infant or preschooler, and an agreement between evaluators greater than 70% was required in at least five consecutive cases to complete the calibration and analyze the other cases.

In each age group, 100 percent of the sample was used to test inter-rater agreement, and 20 to 30 percent of the sample was used to test intra-rater agreement (39 cases selected randomly). To avoid the memory effect, re-evaluations (retests) by the same evaluator were performed at a minimum of 15 days after the initial evaluation.

The Intraclass Correlation Coefficient - ICC was used in the reliability analysis^(2)^ to assess inter- and intra-examiner agreement, classifying it as poor (less than 0.4), fair to good (between 0.4 and 0.7), and excellent (greater than 0.7)^(13)^. In some cases, calculating the ICC was impossible because all individuals in a test displayed the same pattern, with only the percentage of agreement being calculated. The R Core Team 2019 software was used, and the significance level was set at 5%.

## RESULTS

The Orofacial Myofunctional Clinical Examination Protocol with Scores (Appendix 1) was considered, which, along with the Instruction and Clinical History protocols, forms the “MMBGR Protocol – Infants and Preschoolers,” which is appropriate for orofacial myofunctional examinations between the ages of 6 and 71 months.

The following adaptations were initially adopted and made by the researcher with the participation of the authors of the original MBGR instrument during the Content and Appearance Test validation stage of the Orofacial Myofunctional Clinical Examination:

1. Addition: In title: the terms “Infants and Pre-Schools”, as well as the letter “M” of the researcher's surname (Medeiros); in the item Identification: responsible and mother's name; in the item “dentition: deciduous”; in Occlusion: “Functional Maxillary Orthopedics”; “Utensils used in food”; “Suction”; “Pasty Swallowing”; “Solid/Semi-Solid Swallowing” (food used, tongue movement); in speech: “table with chronology of occurrence of the phones”, adequacy of the term “articulatory” precision. There was also the addition of information about which registration should be done according to age group (in months). The items “Suction (breast and baby bottle) and “Pasty” Swallowing were added to the Image Registration Guide.
2. Modifications: The age groups regarding the evaluation of the functions “Suction/Swallowing”, “Chewing”, “Pasty Swallowing” and “Speaking” were revised.
3. Exclusions: Removal of aspects that are not relevant or difficult to register in the age group addressed, such as body posture, measurements of the face, mandibular movements and occlusion; extraoral exam of the face (lateral norm); Masseter (recruitment in isometric contraction); “Mandible” (tooth clenching); “tongue” (brand of device in the language); “teeth” (dental failure and use of prosthesis); “occlusion” (Angle classification and disocclusion guide); “Mobility”; “Sensitivity”; “Breath” (type); “Chewing” (information obtained from the patient's report); “Swallowing” (directed and information obtained through the patient's report); “Speech” (automatic; motor speech coordination; velopharyngeal function); “Voice” (emission of the sustained vowel).

With the assistance of a design professional from the University of São Paulo (USP), a board with illustrative figures (Appendix 2) was also created to be used in the speech assessment - naming test, containing Portuguese-language headphones, preferably in the initial position in the word. This material was created based on a study of the acquisition and occurrence of Portuguese language phones by age group, with the framework of the phoneme acquisition schedule organized, which became part of the new protocol.

At the test content validation stage, most items in the new clinical examination protocol were deemed adequate, with 90.5 percent of agreement and 100 percent of the scores deemed adequate by at least 90 percent of the experts (Table 1). The new protocol was presented to the experts in the second round, and it already included the suggestions made in the first round. At least 70% of respondents said, “I completely agree”.

**Table 1:** Percentage of agreement between evaluators and Content Validity Index regarding specific data of the MMBGR Orofacial Myofunctional Clinical Examination Protocol

**N. of Experts who agree on the application of the MMBGR protocol**	**N. of items (%)**	**CVI (%)**	**p-value**	**N. of Scores (%)**	**CVI (%)**	**p-value**
10	79 (57.7)	100	1.000	75 (75.0)	100	1.000
9	45 (32.8)	90	0.972	25 (25.0)	90	0.972
8	9 (6.6)	80	0.851	0 (0.0)	80	0.851
7	2 (1.5)	70	0.617	0 (0.0)	70	0.617
6	2 (1.5)	60	0.350	0 (0.0)	60	0.350

Exact Binomial Test

**Caption:** CVI= Content Validity Index; % = percentages

The difficulty in obtaining the domain referring to Tone was evident from the data collection method used in the research during the validation step, based on evidence of validity based on the response processes (passive analysis of the edited images). However, for the other domains, the analysis of image reliability revealed inter and intra-observer agreement^(13)^, both in a grouped and more stratified manner (Table 2). The sums of the scores assigned to each item examined in the protocol were taken into account.

**Table 2:** Analysis of inter- and intra-rater agreement for the application of the MMBGR Orofacial Clinical Myofunctional Examination Protocol - grouped and stratified by age group, in months

**Items**	**Inter-evaluator**				**Intra-evaluator**			
	**6-11**	**12-23**	**24-35**	**36-71**	**6-11**	**12-23**	**24-35**	**36-71**
	**ICC**	**ICC**	**ICC**	**ICC**	**ICC**	**ICC**	**ICC**	**ICC**
**Extraoral Exam**	0.62	0.72	0.26	0.52	0.73	0.30	0.79	0.87
FACE	0.37	0.32	0.13	0.27	0.56	-0.13	0.75	0.72
Lips	0.66	0.81	0.59	0.80	0.74	0.33	0.83	0.56
Mandible	0.94	0.79	0.72	0.72	0.82	0.44	0.81	1.00
**Intraoral Exam**	0.26	0.51	0.75	0.39	0.59	0.86	0.88	0.65
Lips	0.25	0.29	0.62	0.75	0.40	0.06	0.89	0.71
Cheeks	0.94^¥^	0.89^¥^	0.81	0.55	1.00^¥^	1.00^¥^	0.96	0.14
Tongue/Fixation	0.37	0.36	0.83	0.60	0.40	0.70	0.90	0.25
Palate	0.03	0.42	0.65	0.31	0.97	0.00	0.89	0.60
Palatine Tonsils	1.00^£^	0.88	0.73	0.47		1.00^£^	1.00	0.94
Teeth and Occlusion	0.94^¥^	0.77	0.64	0.60	1.00^¥^	0.79	0.62	0.90
**Tone**	0.39	0.30	0.13	0.34	0.64	0.72	0.40	0.75
**Breathing**	0.63	0.62	0.40	0.75	0.61	0.15	0.48	1.00
**Suction/Swallowing**	0.12				0.09			
**Chewing**		0.02	0.56	0.45		0.17	0.38	0.71
**Swallowing**	0.82	0.43	0.62	0.49	0.78	0.73	0.51	0.92
**Speech**			0.44	0.65			0.88	0.80

¥ agreement percentage;

**£** insufficient number to calculate ICC or percent agreement

**Caption:** ICC = Intraclass Correlation Coefficient

## DISCUSSION

The study's goal was to present the Orofacial Myofunctional Clinical Examination Protocol from the MMBGR Protocol - Infants and Preschool Children, as well as its adaptation and validation. Initially, evidence of validity was obtained based on the content of the test, which was modified from the MBGR Protocol for use with children aged 6 to 71 months.

The final version of the Protocol was completed based on the authors' professional practice experience, the consulted bibliographic reference, and the experts' approval.

Items that were difficult to record in the age group addressed were excluded from the MMBGR protocol, Clinical Myofunctional Orofacial Examination, such as those that depended on performance through meeting the examiner's order, body posture, measurements of the face, mandibular movements, and occlusion. It was discovered that another instrument, OMES^(9)^, does not measure facial measurements either. On the other hand, based on the study of the chronology of tooth eruption, the item of primary dentition was added, which was relevant for the population studied^(14)^. “Utensils used in food”; “Suction”; “Pasty Swallowing”; and “Solid/Semi-Solid Swallowing” were also added. The content on food development, with standards for age group and skills, was based on the Brazilian Ministry of Health's dietary guide for children under two years old^(15)^, as well as international protocols^(16,17)^.

Aspects of the breastfeeding and complementary feeding pattern, such as the use of artificial teats and suction assessment, were based on the researcher's own work^(3,18)^ as well as the Ministry of Health of Brazil's reference manuals^(19-21)^.

Contents related to Communication and Speech, such as the “table with the chronology of the occurrence of the phones” and the elaboration of the “Figure board” for the naming test, were influenced by studies on Speech Development, particularly in existing language assessment protocols - ABFW - child language test in the areas of phonology, vocabulary, fluency and pragmatics^(22)^ and PROC: behavioral observation protocol: assessment of children's language and cognitive aspects^(23)^. Aspects of articulatory production related to Orofacial Motricity were highlighted at a young age.

The agreement values obtained in the test content validation of the Orofacial Clinical Myofunctional Examination Protocol test are positive, which is consistent with other studies with instruments in the area of Orofacial Motricity that used CVI calculation^(3)^.

The values obtained in the validation step based on the response and reliability processes can also be considered positive, as the vast majority of Extraoral Exam, Intraoral Exam, and Chewing items obtained agreement classified as reasonable to good, or even Excellent. It is worth noting that all age groups had values above 0.4 for the items Breathing, Swallowing, and Speech.

It is worth noting that, for certain domains where agreement was poor in some age groups, the items showed relatively high agreement between 60 and 90 percent in other age groups; however, depending on the number of items, the level of intra-item disagreement, and the level of dependency between the items, the domain score can present many disagreements due to error propagation, that is, the sum of the errors of the combined items greatly increased.

Some considerations should be made regarding the difficulty of obtaining satisfactory agreement between raters for some items in the validation based on evidence of validity based on the response and reliability processes, especially since it is a clinical evaluation protocol that can be applied directly to the patient. However, for this study, it was analyzed using images (static and dynamic).

The item “Tone” demonstrated poor inter-evaluator agreement across all age groups studied, highlighting the difficulty of validating this aspect using the method used (passive analysis of the edited images). The analysis of the Tone through observation of the structures, with their respective mobility, direct palpation, and performance of stomatognathic functions^(24)^, is considered essential in the clinical evaluation.

In all age groups studied, the Extraoral Exam – Face item also demonstrated poor inter-rater agreement. However, a detailed examination of the sub-items revealed that agreement was lower than 70% for some scores. The difficulty of analyzing facial symmetry and proportion without using objective anthropometric criteria, which are important in the accuracy of diagnosis in the area of Orofacial Motricity, is considered^(25)^.

The low agreement values for intraoral exams at young ages correspond with the fact that most infants cannot have an oropharyngeal examination due to crying and stress. The tongue/fixation assessment was carried out with some ease, but the image recording did not always show the precise region of insertion and elevation of the tip of the tongue. Regarding the Suction/Swallowing function, despite the poor agreement, the various aspects obtained high percentages of inter-rater agreement (all above 74.3 percent), with the only difficulty being in classifying the infant's behavioral state at the start of the feeding.

The main difficulty regarding the values of poor agreement between the evaluators in the age group of 12 to 23 months was in Chewing, referring to the observation of the Chewing Pattern - unilateral/bilateral; Food Escape; and Unexpected Muscle Contractions. The infant chewing pattern, which is inherent in normal developmental physiology, is thought to have sparked debate in the study. The introduction of solid food is critical at this age, but there is a gradual process of change in food acceptance, with different textures and flavors being explored between the ages of twelve and twenty-four months^(26)^. Thus, despite the fact that chewing can already be assessed in infants aged 12 to 23 months, the MMBGR Orofacial Clinical Myofunctional Examination Protocol obtained good reliability for the Chewing function only after 24 months, i.e. for preschoolers.

The main difficulties regarding the values of poor agreement between the evaluators were only in the items Face and Tone in the age group from 24 to 35 months, as in other age groups. The main difficulty in the age group of 36 to 71 months was the Intraoral Exam: Palate, which may have occurred due to the analysis of a single image for this item. It is assumed that the analysis based on the direct examination with the patient takes into account the observation and understanding of other aspects, such as dental occlusion conditions, tongue posture observation, and breathing mode. It is regarded as a critical item that must be preserved in the MMBGR Protocol.

The MMBGR Protocol - Brazilian Infants and Preschoolers was developed following the development patterns of the Brazilian Portuguese-speaking population. The use for another population needs cross-cultural validation. New studies aimed at the next steps of validation, such as the criterion and construct validation of the new instrument presented here, are critical.

Finally, it is believed that the presented instrument fills an important gap for the clinic of Orofacial Motricity and its research, thereby expanding scientific knowledge in Speech Therapy.

## CONCLUSION

This article describes the adaptation and validation of the Orofacial Myofunctional Clinical Examination, which is part of the MMBGR Protocol - Infants and Preschoolers, allowing the new instrument to be used for the age group of 6 to 71 months of life, which was previously not covered by specific protocols in OM.

For most items analyzed, the Orofacial Clinical Myofunctional Examination protocol, which incorporates the MMBGR protocol - Infants and Preschoolers, proved to be valid in test content, response processes, and reliability for infants and preschool children without complaints of myofunctional disorders.

## Appendix 1 MMBGR PROTOCOL–Infants and Preschoolers: CLINIC EXAMINATION

MMBGR PROTOCOL

OROFACIAL MYOFUNCTIONAL EXAMINATION WITH SCORES

INFANTS AND PRESCHOOLS (6 months to 5 years and 11 months)

Andréa Monteiro Correia Medeiros, Irene Queiroz Marchesan, Katia Flores Genaro, Giédre Berretin-Felix

1. IDENTIFICATION

**Table:**

Name: ______________________________________________________________		N°: __________________
Exam Date: _____ / _____ / _____	Age: ____years and ___ months	BD: _____ / _____ / _____
Body weight: ________kg	Body height: _______ m	BMI: ___ (weight [kg]/height [m]^2^)
Responsible: ____________________	Mother/father's name:___________________________________________________	

**2. EXTRAORAL EXAM** [ ] Sum of face, lips and mandible scores *(best result = 0 and worst = 20*)

**Face** [ ] Sum of scores (*best result = 0 and worst = 10*) Subjective facial analysis in frontal norm

**Table:**

	**Symmetric**	**Asymmetric**	***Describe***
**Infraorbital plan**	(0)	(1)	
**Zygomatic region**	(0)	(1)	
**Nose wings**	(0)	(1)	
**Cheeks**	(0)	(1)	
**Nasolabial sulcus**	(0)	(1)	
**Upper lip**	(0)	(1)	
**Lip commissure**	(0)	(1)	
**Lower lip**	(0)	(1)	
**Mental**	(0)	(1)	
**Mandible (body and branch)**	(0)	(1)	

**Lips** [ ] Sum of scores (*best result = 0 and worst = 9)*

**Table:**

**Usual posture:**		(0) closed		(1) closed with tension	(2) now open, now closed
		(2) ajar		(2) closed in dental contact	(3) open
**Shape:**	▪ Superior:	(0) normal (1st bow of cupid)		(1) on a gull's wing (Cupid's 1st and 2nd bows)	
	▪ Inferior:	(0) normal		(1) with light eversion	(2) with accentuated eversion
**External mucosa:**		(0) normal	(1) with saliva	(1) parched	(2) wound

**Mandible** [ ] Sum of scores (*best result = 0 and worst = 1*)

**Table:**

**At rest:**	(0) high	(1) lowered

*Observation (Extraoral Exam):* _____________________________________________________________________________________

**3. INTRAORAL EXAM** [ ] Sum of scores of lips, cheeks, tongue, palate, palatines tonsils, teeth and occlusion

(*best result = 0 and worst = 42*): *up to 23 months of age*

(best result = 0 and worst = 56): from 24 months of age

**Lips** [ ] Sum of scores (*best result = 0 and worst = 5*)

**Table:**

**Internal mucosa:**	(0) normal		(1) with dental marks		(2) wounded
**Upper frenulum:**	▪ Fixation to the alveolar ridge:			(0) adequate	(1) low
	▪ Thickness:	(0) adequate		(1) changed: ___________________________________________________	

Observation: __________________________________________________________________________________________________

**Cheeks** [ ] Sum of scores (*best result = 0 and worst = 6): up to 23 months of age*

(*best result = 0 and worst = 8): from 24 months of age*

**Table:**

❒ *Assess in infants (up to 23 months of age)*				
**Mucous:**	(0) normal	(1) oral moniliasis (“thrush”) R	(2) wounded R	
		(1) oral moniliasis (“thrush”) L	(2) wounded L	
❒ *Assess in preschools (from 24 months of age)*				
**Mucous:**	(0) normal	(1) dental marks/apparatus R	(1) alba line R	(2) wounded R
		(1) dental marks/appliance L	(1) alba line L	(2) wounded L

Observation: __________________________________________________________________________________________________

**Tongue** [ ] Sum of scores (*best result = 0 and worst = 13): up to 23 months of age*

(*best result = 0 and worst = 16): from 24 months of age*

**Table:**

❒ *Assess in infants (up to 23 months of age)*						
**Usual posture:**	❒ not visible			(1) compressed in the oral cavity		
	(0) contained in the oral cavity			(1) interposed between teeth and/or gingival ridges		
**Mucous:**	(0) normal		(1) geographic	(1) with cracks	(2) wounded (region):________________________	
❒ *Assess in preschools (from 24 months of age)*						
**Usual posture:**	❒ not visible			(1) on the floor	(1) low point and high back	(2) interdental
**Mucous:**	(0) normal	(1) geographic		(1) with cracks	(2) wounded (region): ________________________	
	(1) marked by teeth (region): ___________________				(1) marked by device (region): ___________	

Observation: __________________________________________________________________________________________________

Frenulum

**Table:**

**Fixation**	▪ on the floor, visible from:		(0) the caruncles	(1) the alveolar crest
	▪ in the tongue:	(0) in the middle third	(1) between the middle third and the apex	(2) at the apex
**Apex shape when lifting tongue:**		(0) rounded	(1) square or rectangular	(2) heart shape
			(1) slight crevice at the apex	(3) does not rise
**Other features:**		(0) none	(1) submucosal or posterior	(2) thick

Observation: __________________________________________________________________________________________________

**Palate** [ ] Sum of scores (*best result = 0 and worst = 10)*

**Table:**

**Hard:**		▪ Depth:			(0) adequate		(1) reduced (low)		(2) increased (high)	
		▪ Width:			(0) adequate		(1) increased (wide)		(2) reduced (narrowed)	
**Palatine veil:**			▪ Symmetry:			(0) present	(1) absent (describe): ______________________________________________			
			▪ Extension:			(0) adequate	(1) long		(2) short	
**Uvula:**	▪ Aspect:			(0) adequate			(1) long	(1) hypoplastic	(1) grooved	(2) bifid

Observation: __________________________________________________________________________________________________

**Palatine tonsils** [ ] Sum of scores (*best result = 0 and worst = 4*)

**Table:**

**Presence:**	❒ present	❒ removed	❒ not visible
**Size:**	(0) adequate	(1) hypertrophy R	(1) hypertrophy L
**Coloring:**	(0) adequate	(1) hyperemia R	(1) hyperemia L

Observation: __________________________________________________________________________________________________

**Teeth and Occlusion** [ ] Sum of scores (*best result = 0 and worst = 4): up to 23 months of age*

(*best result = 0 and worst = 13): from 24 months of age*

**Table:**

**Teeth:**	▪ Upper arch: right ____						left ____		▪ Lower arch: right____		left: ____	
**Oral health:**	▪ Teeth:				(0) good			(1) regular	(2) bad			
	▪ Gums:				(0) good			(1) regular	(2) bad			
❒ *Assess in preschools (from 24 months of age)* ** Evaluate this item/subitem only when there is complete primary dentition, with the presence of second molars.*												
**Medium line:**			(0) adequate					(1) deviated R	(1) deviated L			
**Transversal relation*:**			(0) adequate					(1) posterior crossbite R		(1) posterior crossbite L		
**Horizontal relation:**			(0) adequate					(1) overhang	(1) anterior crossbite			(1) edge to edge bite
**Vertical relation:**			(0) adequate					(1) overbite	(1) posterior open bite R*			
			(1) edge to edge bite					(1) open bite anterior	(1) posterior open bite L*			
**Relationship between canines *:**				(0) class I R				(1) class II R	(1) class III R			
				(0) class I L				(1) class II L	(1) class III L			
**Device use:**		❒ no				❒ yes: Type: __________________________________________________________________						

Observation: __________________________________________________________________________________________________

**4. TONE** [ ] Sum of scores *(best result = 0 and worst = 6)* (*perform visual observation and palpation*)

**Table:**

	**Normal**	**Decreased**	**Increased**
**Upper lip:**	(0)	(1)	(1)
**Lower lip:**	(0)	(1)	(1)
**Mental:**	(0)	(1)	(1)
**Tongue:**	(0)	(1)	(1)
**Cheek R:**	(0)	(1)	(1)
**Cheek E:**	(0)	(1)	(1)

Observation: __________________________________________________________________________________________________

**5. OROFACIAL FUNCTIONS** [ ] Sum of scores from breathing, suction, chewing, swallowing and speech

**Breathing** [ ] Sum of scores *(best result = 0 and worst = 2)*

If changed, it relates to: [ ] habit [ ] possible obstructive factor [ ] other: ____________________

**Table:**

**Mode:**	(0) nasal	(1) oronasal	(2) oral
**Nasal flow** *(use the mirror)*:		❒ similar between the nostrils	❒ asymmetry: [ ] mild [ ] moderate [ ] accentuated

Observation: __________________________________________________________________________________________________

**Suction/Swallowing** [ ] sum of scores *(best result = 0 and worst = 22)*

**Table:**

❒ *Assess up to 23 months of age, in infants who are still breastfeeding (breastfeeding) or using a baby bottle*																	
**Food supply route:**		[ ] breast				[ ] baby bottle (describe the type of beak: ____________________________________________											
**Liquid used:**		❒ water				❒ milk	❒ juice		❒ other: _________________________________________								
**Behavioral state** (start):						(0) alert	(1) light sleep/sleepy				(1) agitated/irritated					(2) crying	
**Suction pattern:**			(0) present - regular groups					(1) present - irregular groups						(2) sporadic suction			(3) absent
**Suction strength:**			(0) strong			(1) average	(2) weak		(3) absent								
**Lips posture:**			(0) total sealing				(1) partial sealing					(2) unsealed					
**Orbicularis contraction:**				(0) adequate			(1) few		(1) accentuated				(2) absent				
**Mental contraction:**				(0) absent			(1) few		(1) accentuated								
**Tongue movement:**				❒ unobservable			(0) organized		(1) unorganized: _________________________________								
**Head movement:**				(0) absent			(1) present										
**Liquid containment:**				(0) adequate			(1) inadequate, with little escape								(2) inadequate, with a lot of escape		
**Rhythm:**	(0) satisfactory			(1) fast (no breaks)			(1) slow		(2) absent								
**Noise:**	(0) absent			(1) present													
**Coordination: suction/breathing/swallowing:**							(0) adequate			(1) choke				(1) cough			
**Waste after swallowing:**					(0) absent		(1) present										

Observation: __________________________________________________________________________________________________

**Chewing** [ ] sum of scores *(best result = 0 and worst = 13)*

If changed, the origin is: [ ] functional [ ] structural [ ] other: _______________________________

**Solid chew** [ ] *(food containing larger pieces, in the same consistency as the family's diet)*

**Semi-solid chewing** [ ] *(food containing very small, soft or shredded pieces)*

(*If it is the expected standard or the item does not apply for the age, consider zero)*

❒ *Assess from 12 months of age*

**Table:**

**Food used:**				❒ bread			❒ cookie (type):_____________			❒ fruit in pieces			❒ family/school food	
							❒ other: __________________________________________________________________							
**Incision:**	(0) anterior						(1) lateral		(2) does not perform			(1) other: ____________________________		
**Crushing:**			(0) posterior teeth						(0) anterior teeth in the absence of molars					(0) efficient
			(1) tongue kneading						(1) anterior teeth in the presence of molars					(2) inefficient
**Chewing pattern:**					(0) alternate unilateral/bilateral				(1) simultaneous bilateral					
					(0) unilateral preferential				(2) chronic unilateral					
**Lip closure:**					(0) systematic				(1) unsystematic					
**Noisy chewing:**					(0) no		(1) yes							
**Food escape:**					(0) no		(1) yes							
**Unexpected muscle contractions:**								(0) absent			(1) present (describe): _______________________			
**Exacerbated oral reflexes:**						(0) absent			(1) present (gag)			(1) present (bite)		
**Rhythm:**		(0) adequate				(1) slow		(1) fast						

Observation: ______________________________________________________________________________________________

**Swallowing** [ ] sum of scores liquid + pasty ( *best result = 0 and worst = 37)*

sum of scores liquid+ solid + semisolid *(best result = 0 and worst = 32)*

If changed, the origin is: [ ] functional [ ] structural [ ] other: _______________________________

(**if it is the expected standard for the age, consider zero. Note valid for all consistencies)*

**Solid swallowing** [ ] *(food containing larger pieces, in the same consistency as the family's diet)*

**Semisolid swallowing** [ ] *(food containing pieces cut very small and soft are shredded)*

sum of scores *(best result = 0 and worst = 17)*

**Table:**

❒ *Assess from 12 months of age: According to diet acceptance. When already accepting solid, do not evaluate semi-solid*								
**Food used:**				❒ bread	❒ cookie (type): __________	❒ fruit in pieces	❒ family/school food	
				❒ Outro: _________________________________________________________________________________				
**Utensils used in food:**				❒ hands	❒ spoon	❒ fork	❒ other: _________________	
**Readiness:**		(0) present (open mouth food approaches/touches lips)					(1) absent	
**Lips posture:**				(0) closed	(1) lower lip in contact with upper teeth		(2) opened	
					(1) partially closed			
**Tongue posture *:**				❒ unobservable	(0) behind the teeth	(1) against teeth	(2) between teeth	
**Tongue movement *:**				❒ unobservable	(0) anteroposterior	(1) kneading	(1) posteroanterior	(2) absent
**Food containment:**				(0) adequate	(1) partial	(2) inadequate - with escape		
**Orbicularis contraction:**				(0) adequate	(1) few	(2) accentuated		
**Mental contraction:**				(0) absent	(1) few	(2) accentuated		
**Head movement:**				(0) absent	(1) present			
**Rhythm*:**	(0) one swallow				(1) two swallows	(2) multiple swallows		
**Noise:**	(0) absent			(1) present				
**Coordination:**			(0) adequate		(1) choke	(1) cough		
**Waste after swallowing:**				(0) absent	(1) present			

Observation: __________________________________________________________________________________________________

**Pasty swallowing** *(porridge, puree/mashed food)* [ ] sum of scores *(best result = 0 and worst = 22)*

**Table:**

❒ *evaluate up to 11 months of age: (you can evaluate up to 23 months, in infants that feed in the pastry consistency)*																				
**Food used:**				❒ porridge				❒ puree		❒ *mashed food* (what): _______________________________________________										
**Utensils used in food:**					❒ spoon			❒ other: __________________________________________________________________												
**Readiness:**		(0) present *(open mouth when spoon approaches/touches lips)*													(1) absent					
**Bite reflex:**					(0) present					(1) exacerbated					(1) absent					
**Gag reflex:**					(0) present					(1) exacerbated					(1) absent					
**Lip posture:**					(0) closed					(1) lower lip in contact with upper teeth									(2) opened	
										(1) partially closed										
**Lip movement:**						(0) adequate *(move upper lip to remove food from spoon)*									(1) few (exaggerated)			(1) exaggerated		
**Tongue posture*:**					❒ unobservable					(0) behind the teeth				(1) against teeth			(2) between teeth *****			
**Tongue movement *:**						❒ unobservable				(0) anteroposterior		(1) kneading *****				(1) posteroanterior				(2) absent
**Food volume:**						(0) satisfactory				(1) increased		(1) decreased								
**Food containment:**							(0) adequate			(1) inadequate – com escape										
**Orbicularis contraction:**							(0) adequate			(1) few		(2) accentuated								
**Mental contraction:**							(0) absent			(1) few		(2) accentuated								
**Head movement:**							(0) absent			(1) present										
**Rhythm:**	(0) one swallow									(1) two swallows		(2) multiple swallows								
**Noise:**	(0) absent									(1) present										
**Coordination:**			(0) adequate						(1) choke		(1) cough									
**Waste after swallowing:**							(0) absent			(1) few			(2) a lot							

Observation: __________________________________________________________________________________________________

**Pasty swallowing** *(do not use a bottle to assess)* [ ] sum of points *(best result = 0 and worst = 15)*

**Table:**

❒ *Assess from 12 months of age*								
**Liquid used:**		❒ water				❒ milk	❒ juice	❒ other: ___________________
**Utensils used in food:**				❒ common cup		❒ cup with lid	❒ cup with valve	❒ other: ___________________
**Lip posture:**			(0) closed			(1) lower lip in contact with upper teeth		(2) opened
						(1) partially closed		
**Tongue posture *:**			❒ unobservable			(0) behind the teeth	(1) against teeth	(2) between teeth *****
**Liquid volume:**			(0) satisfactory			(1) increased	(1) decreased	
**Liquid containment:**					(0) adequate	(1) inadequate – with escape		
**Orbicularis contraction:**					(0) adequate	(1) few	(2) accentuated	
**Mental contraction:**					(0) absent	(1) few	(2) accentuated	
**Head movement:**					(0) absent	(1) present		
**Rhythm:**	(0) sequential					(1) sip by sip		
**Noise:**	(0) absent					(1) present		
**Coordination:**				(0) adequate		(1) choke	(1) cough	(1) voice change/wet voice

Observation: __________________________________________________________________________________________________

**Speech** [ ] sum of scores - production of phones / phonemes + general aspects of speech articulation *(best result = 0 and worst = 21)*

Characteristic: [ ] Phonological [ ] Phonetics / Phonological [ ] Phonetics

Se phonetic alteration, the origin is: [ ] functional [ ] structural [ ] neuromuscular [ ] other:_______________

**Production of phones/phonemes** [ ] Sum of scores *(best result = 0 and worst = 6)*

**Table:**

❒ *Assess from 12 months of age:* (0) absent (1) present (*if expected for the age, consider zero)*			
**Figure naming/Repetition** [ ] sum all points *(best result = 0 and worst = 3)* *Use MMBGR Protocol - Figures for Naming* () replacement () omission () distortion			
**semi-directed speech** [ ] sum all points *(best result = 0 and worst = 3)* *Saying name and age / Talking about school or a joke / Telling about a trip or tour* *() replacement () omission () distortion*			
Phones/Phonemes and characteristics: fill in the table below			
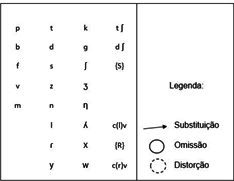
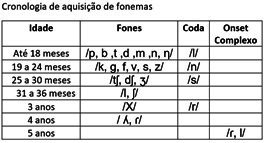
**In case of articulation point replacement:**		[ ] audibly perceptible	[ ] visually noticeable
**In the case of distortion, it relates to the:**	[ ] absence/little vibration of the tip of the tongue		[ ] back elevation
	[ ] multiple tongue apex flutter		[ ] lowering of the back
	[ ] interdental language: () anterior () lateral		

Protocol elaborated following development patterns of the Brazilian Portuguese-speaking population. Use for another population needs cross-cultural validation.

Observation: __________________________________________________________________________________________________

**Therapeutic test** *Request the repetition of syllables containing the altered sounds, combined with the vowel “e”*

Note if there is a change in the issue when the correct model is provided

**Table:**

**Phone tested**	**production does not change**	**production improves**	**the production becomes adequate**
	[ ]	[ ]	[ ]
	[ ]	[ ]	[ ]
	[ ]	[ ]	[ ]

Observation: __________________________________________________________________________________________________

**General aspects of speech articulation** [ ] Sum of points *(best result = 0 and worst = 15)*

**Table:**

❒ *Evaluate from 36 months of age:*													
**Saliva:**		(0) swallowed				(1) accumulated in the right and/or left commissure				(2) sneezes			(3) drool
						(1) accumulated in the lower lip							
**Mouth opening:**				(0) adequate				(1) reduced		(1) increased			
**Tongue position in speech:**						(0) adequate		(1) on the floor		(2) posteriorized			
								(2) interdental (projection)		(2) low apex and high sides			
**Mandible movement:**						(0) adequate		(1) right turn		(1) left turn			(1) anteriorization
**Lips movement:**						(0) adequate		(1) reduced		(1) exaggerated			
**Tongue movement:**						(0) adequate		(1) reduced					
**Velocity:**			(0) adequate					(1) increased		(1) reduced			
**Resonance:**			(0) oral balance					(1) reduced nasal use: () mild		() moderate		() severe	
			(1) laryngopharyngeal					(1) nasal overuse: () mild		() moderate		() severe	
**Pneumophonoarticulatory coordination:**								(0) adequate		(1) changed ________________________________			
**Articulation:**			(0) precise					(1) unsystematic imprecision		(2) systematic imprecision			
In the event of inaccuracy, it is related to:													
[ ]tone [ ] hearing			[ ]speech speed [ ]oral breathing				[ ]amount of saliva [ ]malocclusion		[ ]muscle fatigue [ ]mouth opening reduction		[ ]neurological disorder [ ]outher:_______________________		
**Voice**	*▪ Pitch:*				[ ]Adequate		[ ]Low		[ ]High				
	*▪ Loudness:*				[ ]Adequate		[ ]Strong		[ ]Weak				
	*▪* Tipo:				[ ]Adequate		[ ]Altered						

Observation: __________________________________________________________________________________________________

_____________________________________________________________________________________________________________

**Table:**

**Script for registration of images**						
**Static Images**						
- Face:	[ ] Frontal view without head posture correction				[ ] Front view with corrected head posture	
- Lips:	[ ] At rest - usual		[ ] Internal mucosa		[ ] Superior labral frenulum	
- Cheeks:	[ ] Right internal mucosa		[ ] Left internal mucosa			
- Tongue:	[ ] Externalized *(out of the oral cavity)*					
	[ ] Frenulum *(tongue raised without touching the palate)*				[ ] Frenulum *(high tongue with maneuver)*	
- Palate:	[ ] Hard					
- Teeth:	[ ] Upper arcade		[ ] Lower arcade			
- Occlusion:	[ ] Anterior		[ ] Right side		[ ] Left side	
- Others:	[ ] At the discretion of the examiner					
**Dynamic Images**						
- Suction:	[ ] Breastfeeding (breast)			[ ] Baby Bottle		
- Chewing:	[ ] Open mouth after chewing and before swallowing					
- Swallowing:	[ ] Liquid	[ ] Pasty		[ ] Solid/Semi-solid		[ ] Open mouth after swallowing *(residue)*
- Speech:	[ ] Semi-directed	[ ] Figure naming/repetition				
- Oropharynx:	[ ] Soft palate	[ ] Uvula		[ ] Palatine tonsils		

**Table:**

**Data collected from exams: __________________________________________________________________**				
__________________________________________________________________________________________				
__________________________________________________________________________________________				
__________________________________________________________________________________________				
__________________________________________________________________________________________				
__________________________________________________________________________________________				
**Requested exams (justification): ______________________________________________________________**				
__________________________________________________________________________________________				
__________________________________________________________________________________________				
__________________________________________________________________________________________				
__________________________________________________________________________________________				
__________________________________________________________________________________________				
**Speech therapy diagnosis: __________________________________________________________________**				
__________________________________________________________________________________________				
__________________________________________________________________________________________				
__________________________________________________________________________________________				
__________________________________________________________________________________________				
__________________________________________________________________________________________				
__________________________________________________________________________________________				
**Prognosis:**	[ ] favorable	[ ] limited	[ ] unfavorable
__________________________________________________________________________________________				
__________________________________________________________________________________________				
__________________________________________________________________________________________				
**Referral to other professionals (area and justification): ___________________________________________**				
__________________________________________________________________________________________				
__________________________________________________________________________________________				
__________________________________________________________________________________________				
__________________________________________________________________________________________				
**Therapeutic plan: ___________________________________________________________________________**				
__________________________________________________________________________________________				
__________________________________________________________________________________________				
__________________________________________________________________________________________				
__________________________________________________________________________________________				
__________________________________________________________________________________________				
__________________________________________________________________________________________				
__________________________________________________________________________________________				

Summary of the Orofacial Myofunctional Exam - mmbgr - Infants and Preschools

Andréa Monteiro Correia Medeiros, Irene Queiroz Marchesan, Katia Flores Genaro, Giédre Berretin-Felix

**Table:**

**EXTRAORAL EXAM - Age group (months/year)**	**06/nov**	**dez/23**	**24-35**	**36-71**
		**(1 year)**	**(2 years)**	**(3-5 years)**
**(best result = 0 and worst = 20)**	**[ ]**	**[ ]**	**[ ]**	**[ ]**
	**0-20**	**0-20**	**0-20**	**0-20**
Face (*best result = 0 and worst = 10)*	[ ]	[ ]	[ ]	[ ]
Lips (*best result = 0 and worst = 9)*	[ ]	[ ]	[ ]	[ ]
Mandible (*best result = 0 and worst = 1)*	[ ]	[ ]	[ ]	[ ]
**INTRAORAL EXAM**	**[ ]**	**[ ]**	**[ ]**	**[ ]**
**(best result = 0 and worst = 42/56)**	**0-42**	**0-42**	**0-56**	**0-56**
Lips *(best result = 0 and worst = 5)*	[ ]	[ ]	[ ]	[ ]
Cheeks *(best result = 0 and worst = 6/8)*	[ ]	[ ]	[ ]	[ ]
Tongue *(best result = 0 and worst = 13/16)*	[ ]	[ ]	[ ]	[ ]
Palate *(best result = 0 and worst = 10)*	[ ]	[ ]	[ ]	[ ]
Palatine tonsils *(best result = 0 and worst = 4)*	[ ]	[ ]	[ ]	[ ]
Teeth and occlusion *(best result = 0 and worst = 4/13)*	[ ]	[ ]	[ ]	[ ]
**TONE**	**[ ]**	**[ ]**	**[ ]**	**[ ]**
**(best result = 0 and worst = 6)**	**0-6**	**0-6**	**0-6**	**0-6**
Lips *(upper+lower) (best result = 0 and worst = 2)*	[ ]	[ ]	[ ]	[ ]
Mental *(best result = 0 and worst = 1)*	[ ]	[ ]	[ ]	[ ]
Tongue *(best result = 0 and worst = 1)*	[ ]	[ ]	[ ]	[ ]
Cheeks *(right+left) (best result = 0 and worst = 2)*	[ ]	[ ]	[ ]	[ ]
**OROFACIAL FUNCTIONS**	**[ ]**	**[ ]**	**[ ]**	**[ ]**
**(best result = 0 and worst = 46/92/53/68)**	**0-46**	**0-92**	**0-53**	**0-68**
Breathing *(best result = 0 and worst = 2)*	[ ]	[ ]	[ ]	[ ]
Suction/Swallowing *(best result = 0 and worst = 22)*	[ ]	[ ]	___	___
Chewing *(best result = 0 and worst = 13)*	___	[ ]	[ ]	[ ]
Swallowing Liquid+ pasty (*best result = 0 and worst = 37)*				
Swallowing Liquid + solid/semi-solid *(best result = 0 and worst = 32)*				
Swallowing semi-solid/solid *(best result = 0 and worst = 17)*	___	[ ]	[ ]	[ ]
Swallowing pasty *(best result = 0 and worst = 22)*	[ ]	[ ]	___	___
Swallowing liquid *(best result = 0 and worst = 15)*	___	[ ]	[ ]	[ ]
Speech (best result = 0 and worst = 6/21)				
Production of phones/phonemes *(best result = 0 and worst = 6)*	___	___	[ ]	[ ]
General aspects of speech articulation *(best result = 0 and worst = 15)*	___	___	___	[ ]
**TOTAL SCORE**	**[ ]**	**[ ]**	**[ ]**	**[ ]**

Speech therapist: ____________________________________ CRFª: ___________

## Appendix 2 Board (Front and Back) – Figures for Nomination – MMBGR Protocol – Infants and Preschoolers

Andréa Monteiro Correia Medeiros, Irene Queiroz Marchesan, Katia Flores Genaro, Giédre Berretin-Felix

Protocol elaborated following development patterns of the Brazilian Portuguese-speaking population. Use for another population needs cross-cultural validation.
