# ERRATUM: MMBGR Protocol - infants and preschoolers: myofunctional orofacial clinic examination

**DOI:** 10.1590/2317-1782/20212022190en

**Published:** 2023-01-06

Due to author's honest mistake the article “MMBRG Protocol - infants and preschoolers: myofunctional orofacial clinic examination” (DOI https://doi.org/10.1590/2317-1782/20212020325), published in CoDAS 2022;34(5):e20200325, was published with errors.

**On Portuguese title, where the text reads:**

Protocolo MMBRG - lactentes e pré-escolares: exame clínico miofuncional orofacial

**It should read:**

Protocolo MMBGR - lactentes e pré-escolares: exame clínico miofuncional orofacial

**On English title, where the text reads:**

MMBRG Protocol - infants and preschoolers: myofunctional orofacial clinic examination

**It should read:**

MMBGR Protocol - infants and preschoolers: myofunctional orofacial clinic examination

**On English version on pages 3, 4, 10, 11, 13 and 15, where the text reads:**

Pasty

**It should read:**

Pudding

**On English version on page 11, in the first chart “Pasty swallowing” where the text reads:**

**Lip movement:** (0) adequate *(move upper lip to remove food from spoon)* (1) few (exaggerated) (1) exaggerated

**It should read:**

**Lip movement:** (0) adequate *(move upper lip to remove food from spoon)* (1) few (1) exaggerated

**On English version on page 11, in the second chart where the text reads:**

**Pasty swallowing** (do not use a bottle to assess)

**It should read:**

**Liquid swallowing** (do not use a bottle to assess)

The authors apologize for the errors.

