# Supplementary material for: MMBRG Protocol – Infants and Preschoolers: Myofunctional Orofacial Clinic Examination
Source: Codas. 2022 Jun 17;34(5):e20200325. doi: 10.1590/2317-1782/20212020325 (PMC9886187; doi:10.1590/2317-1782/20212020325)
Supplement: Translation

# Translation: Protocolo MMBRG – Lactentes e Pré-Escolares: Exame Clínico Miofuncional Orofacial

**Authors:** Andréa Monteiro Correia Medeiros, Irene Queiroz Marchesan, Katia Flores Genaro, Íkaro Daniel de Carvalho Barreto, Giédre Berretin-Felix

**Affiliations:** 1 Departamento de Fonoaudiologia, Universidade Federal de Sergipe – UFS - São Cristóvão (SE), Brasil.; 2 CEFAC Saúde e Educação – São Paulo (SP), Brasil.; 3 Departamento de Fonoaudiologia – Faculdade de Odontologia de Bauru – FOB, Universidade de São Paulo – USP - Bauru (SP), Brasil; 4 Programa de Pós-graduação em Biometria e Estatística Aplicada, Universidade Federal Rural de Pernambuco – UFRPE - Recife (PE), Brasil.

**Keywords:** Fonoaudiologia, Lactente, Pré-escolar, Estudos de Validação, Terapia Miofuncional, Sistema Estomatognático

## Abstract

**Objetivo:**

Apresentar Exame Clínico Miofuncional Orofacial pertencente ao Protocolo MMBGR - Lactentes e Pré-escolares, incluindo sua validação.

**Método:**

Inicialmente foi realizada a validade do conteúdo do teste adaptado do Protocolo MBGR, para faixa etária entre 6 e 71 meses, fundamentada na bibliografia e experiência entre autores (originais e atuais). Para análise de conteúdo e aparência participaram 10 fonoaudiólogos especialistas em Motricidade Orofacial, que preencheram formulário eletrônico com questões dicóticas e escala de Likert, em dois momentos. Foi calculado Índice de Validade de Conteúdo e Teste Binomial Exato. Na sequência houve análise da validade baseada nos processos de resposta, seguida da análise da confiabilidade do Exame Clínico, com 155 participantes, por 7 fonoaudiólogos experientes e calibrados, sendo verificada a concordância entre e intra examinadores pelo Coeficiente de Correlação Intraclasse.

**Resultados:**

Houve acréscimos, modificações e exclusão de itens conforme faixa etária, concluindo-se o Protocolo Exame Clínico Miofuncional Orofacial para lactentes e pré-escolares, que obteve 90,5% com concordância; e 100% dos escores adequados por pelo menos 90% dos especialistas. Quanto à confiabilidade, a maioria dos itens dos Exames Extraoral e Intraoral e Mastigação obtiveram concordância razoável a boa, ou, até mesmo, excelente.

**Conclusão:**

O “Exame Clínico Miofuncional Orofacial” teve validação baseada no conteúdo do teste, nos processos de resposta e confiabilidade concluída, e junto ao “Instrutivo” e à “História Clínica” integra o “Protocolo MMBGR - Lactentes e Pré-escolares”, para atuação fonoaudiológica na faixa etária entre 6 e 71 meses de idade.

## INTRODUÇÃO

O exame clínico ocupa papel central na clínica fonoaudiológica para estabelecer diagnóstico e prognóstico na área de Motricidade Orofacial (MO). Instrumentos padronizados que contemplem a clínica e a pesquisa, permitem que o fonoaudiólogo planeje, documente e analise a evolução e efetividade do processo terapêutico^(1)^. A validação de testes é importante, conforme parâmetros estabelecidos^(2)^. Especificamente, têm-se preconizado que os estudos de validação contemplem as seguintes etapas, quando se trata de teste de Fonoaudiologia: Evidência de validade baseada em conteúdo, consistência interna e relação com outras variáveis; Evidência de validade baseada nos processos de resposta; Confiabilidade/Precisão; Equidade; Acurácia; e respectiva Evidência de validade baseada nas consequências do teste^(2)^.

Considerando a área de MO em aleitamento materno, instrumentos foram desenvolvidos para acompanhar a díade mãe-recém-nascido^(3)^ e avaliar prontidão para mamada em recém-nascidos, incluindo casos de risco^(4-6)^; além de aspectos morfofisiológicos específicos^(7)^. Em outra ponta, protocolos padronizados da avaliação miofuncional orofacial, voltados à população a partir de 6 anos de idade, como o AMIOFE-E^(8,9)^ e o MBGR^(10,11)^, já são amplamente reconhecidos na Fonoaudiologia.

Entretanto, até o momento, não foi encontrada publicação brasileira contendo um instrumento padronizado e validado da área de MO que fosse destinado à faixa etária entre 6 meses e 5 anos e 11 meses de vida, o que se mostrou uma lacuna importante.

Considerando a escassez de instrumentos padronizados voltados para a MO de lactentes e pré-escolares, o objetivo deste trabalho é apresentar a versão final do “Exame Clínico Miofuncional Orofacial”, que compõe o “Protocolo MMBGR – Lactentes e Pré-Escolares”, evidenciando a validação de conteúdo do teste, evidência de validade baseada nos processos de resposta e confiabilidade.

## MÉTODO

Estudo descritivo, integrante de projeto de pesquisa aprovado pelo Comitê de Ética e Pesquisa em Seres Humanos da Universidade Federal de Sergipe, sob nº CAEE 12529419.6.0000.5546. Todos os participantes e/ou responsáveis assinaram o Termo de Consentimento Livre e Esclarecido (TCLE). Trata-se da validação de um novo instrumento voltado à população de lactentes e pré-escolares, adaptado a partir do protocolo MBGR^(11)^, conforme diretrizes dos estudos de validação^(2)^, após obter-se parecer escrito favorável à adaptação pelas autoras do protocolo MBGR original^(11)^.

Inicialmente houve a etapa de validade baseada no conteúdo do teste. A partir de estudo teórico e experiência do pesquisador foi organizado o novo instrumento, com revisão e consenso entre autores (versões original e atual). Foi realizada revisão da literatura sobre o desenvolvimento miofuncional orofacial e as funções estomatognáticas em idade precoce, por meio de busca nas plataformas Scielo, Pubmed e Bireme, no período de 1993 a 2017. Utilizados os descritores Fonoaudiologia, Lactentes, Pré-escolares, Métodos de Avaliação e Sistema Estomatognático.

O instrumento passou pela análise da aparência e do conteúdo. Essa etapa contou com 10 fonoaudiólogos especialistas e com experiência na área de MO. Como critério de inclusão, considerou-se: tempo maior que cinco anos de atuação e/ou atividade docente em Fonoaudiologia; ter titulação e/ou publicações relacionadas à área de MO. Como critério de exclusão, a não entrega dos pareceres nos prazos estipulados.

A maioria deles apresentava mais de 15 anos (90%) de atuação, experiência docente há pelo menos 5 anos e atuação com lactentes (80%) e pré-escolares (80%). Esses profissionais residem em 4 regiões do Brasil (Centro-Oeste, Nordeste, Sudeste e Sul); 80% possuem título de Doutor e 20% de Mestrado. A maioria (70%) está na faixa etária entre 41 e 50 anos de idade.

Na validação baseada na análise do conteúdo do teste, utilizou-se formulário eletrônico contendo questões dicóticas (sim/não), com campos para justificar as respostas negativas (descrever o aspecto que não concordava em determinado item, podendo sugerir modificação). Foram calculados o Índice de Validade de Conteúdo (IVC) e aplicado o Teste Binomial Exato, com nível de concordância mínima de 70%. Houve segunda rodada de análise do instrumento, com escala de Likert^(2,12)^ com cinco opções de resposta (concordo plenamente, concordo, indiferente, discordo e discordo totalmente).

Na sequência houve a análise da validade baseada nos processos de resposta, seguida da análise da confiabilidade do Exame Clínico Miofuncional Orofacial, realizada por sete fonoaudiólogas com experiência na avaliação da MO em crianças menores de 6 anos de idade, a partir da análise de imagens padronizadas.

Para tanto, foram registradas imagens do exame clínico individual de crianças, com duração aproximada de 30 minutos, aplicado pela pesquisadora (avaliadora 1). Critérios de inclusão: o lactente e/ou pré-escolar deveria ser saudável, sem acometimentos neurológicos. Critério de exclusão: recusa do menor/responsável para se submeter ao Exame Clínico Miofuncional Orofacial, no todo ou em parte.

Foram recrutados 260 lactentes e pré-escolares, conforme critérios de elegibilidade e assinatura do TCLE. Destes, 46 não aceitaram a avaliação (parcial ou integralmente), e 10 tiveram o registro de imagens incompatível para análise. Dos 204 sujeitos avaliados e com imagens compatíveis, foram considerados 155 lactentes e pré-escolares, 93 (60%) do estado de Sergipe e 62 (40%) do estado de São Paulo, distribuídos nas faixas etárias: 6 a11 meses (N=35); 12 a 23 meses (N=35); 24 a 35 meses (N=35) e 36 a 71 meses (N=50).

A coleta de dados foi realizada em 4 instituições: 2 creches da cidade de Bauru, no interior do estado de São Paulo; 1 creche na cidade de São Cristóvão, estado de Sergipe, e no ambulatório infantil do Hospital Universitário da Universidade Federal de Sergipe em Aracaju, as quais disponibilizaram uma sala para o procedimento.

Padronizou-se para a coleta de dados a posição sentada em cadeira, compatível com a estatura, com os pés da criança apoiados no chão. O lactente geralmente era posicionado no colo do responsável, com as costas e cabeça apoiadas, mantendo-se de frente para o examinador. Em alguns casos de pré-escolares, o procedimento também foi realizado na presença e/ou no colo do professor, do auxiliar da creche, ou do responsável. Foi utilizado fantoche e um brinquedo, para possibilitar um ambiente lúdico e conseguir e atenção e aproximação da criança. Porém, garantiu-se que todos os procedimentos de avaliação fossem cumpridos e registrados de modo padronizado.

O registro das imagens estáticas (JPEG) e dinâmicas (MP4) foi feito por outros dois avaliadores devidamente treinados e calibrados, por meio de câmera digital (Panasonic Compact-VHS Palmcorder) posicionada em mãos, com imagem aproximada da região orofacial (lente Macro Led Ring Flash HD). Usou-se tripé para filmar a prova de nomeação. Tais imagens foram editadas por um grupo de 12 discentes da área da Saúde, a partir de um treinamento prévio proposto pela pesquisadora. Todos os registros foram assistidos pela pesquisadora, conferindo se estavam compatíveis ao preenchimento do novo instrumento de avaliação.

Para a análise da confiabilidade, as imagens editadas foram compartilhadas com 7 fonoaudiólogos avaliadores. O Avaliador 1 (A1) (pesquisadora principal), considerado especialista, analisou todos os casos da pesquisa; enquanto os demais 6 Avaliadores (A) 2, 3, 4, 5, 6 e 7, foram distribuídos por grupo etário: 6 a11 meses (A2); 12 a 23 meses (A3); 24 a 35 meses (A4, A5 ou A6); 36 a 71 meses (A6 ou A7), formando dupla com o A1, configurando um segundo avaliador para análise de cada caso.

Previamente foi realizado o procedimento de Calibração entre os avaliadores, conforme diretrizes de análise de cada aspecto observado, por faixa etária. Após a calibração, cada dupla de avaliadores aplicou o protocolo com o mesmo lactente ou pré-escolar, de forma independente, tendo sido necessário obter concordância entre avaliadores maior que 70%, em ao menos 5 casos consecutivos, para finalização da calibração, seguindo-se para análise dos demais casos.

Para testar concordância inter-avaliadores foi considerada 100% da amostra, e intra avaliador cerca de 20 a 30% da amostra, de cada faixa etária (39 casos selecionados aleatoriamente). As reavaliações (reteste) pelo mesmo avaliador, ocorreu com o intervalo mínimo de 15 dias da avaliação inicial, a fim de evitar efeito de memória.

Na análise da confiabilidade foi aplicado o Coeficiente de Correlação Intraclasse - CCI^(2)^ para avaliar a concordância inter e intra-examinador, classificando-se em pobre (inferior a 0,4), de razoável a boa (entre 0,4 e 0,7) e excelente (maior que 0,7)^(13)^. Em algumas situações não foi possível calcular o CCI devido todos os indivíduos em algum teste apresentarem o mesmo padrão, sendo calculado apenas o percentual de concordância. O nível de significância adotado foi de 5% e o software utilizado foi o R Core Team 2019.

## RESULTADOS

Considerou-se o Protocolo de Exame Clínico Miofuncional Orofacial com escores (Apêndice 1) que compõe, junto ao Instrutivo e o protocolo de História Clínica, o “Protocolo MMBGR – Lactentes e Pré-Escolares”, adequado para o exame miofuncional orofacial entre as faixas etárias de 6 a 71 meses.

Na etapa de validação do Conteúdo do teste e da Aparência do Exame Clínico Miofuncional Orofacial, foram adotadas inicialmente as seguintes adaptações, realizadas pela pesquisadora, com a participação dos autores do instrumento MBGR original:

1. Acréscimo: Em título: os termos “Lactentes e Pré-Escolares”, bem como a letra “M” do sobrenome do pesquisador (Medeiros); no item Identificação: responsável e nome da mãe; no item “dentição: decídua”; em Oclusão: “ortopedia funcional dos maxilares”; “Utensílios utilizados na alimentação”; “Sucção”; “Deglutição de pastoso”; “Deglutição de sólido/semissólido” (alimento utilizado, movimento da língua); em fala: “quadro com cronologia de ocorrência dos fones”, adequação do termo precisão “articulatória”. Também houve o acréscimo da informação sobre qual é o registro a ser feito de acordo com a faixa etária (em meses). No Roteiro para Registro de Imagens foram acrescidos os itens “Sucção (seio materno e mamadeira), e Deglutição “Pastoso”.
2. Modificações: Revisadas as faixas etárias referentes à avaliação das funções “Sucção/Deglutição”, “Mastigação”, “Deglutição de pastoso” e “Fala”.
3. Exclusões: Retirados os aspectos não pertinentes ou de difícil registro na faixa etária abordada, tais como postura corporal, medidas da face, dos movimentos mandibulares e da oclusão; exame extraoral da face (norma lateral); Masseter (recrutamento na contração isométrica); “Mandíbula” (apertamento dentário); “língua” (marca de aparelho na língua); “dentes” (falha dentárias e uso de prótese); “oclusão” (classificação de Angle e guia de desoclusão); “Mobilidade”; “Sensibilidade”; “Respiração” (tipo); “Mastigação” (informações obtidas através do relato do paciente); “Deglutição” (dirigida e informações obtidas através do relato do paciente); “Fala” (automática; coordenação motora da fala; função velofaríngea); “Voz” (emissão da vogal sustentada).

Também foi elaborada, com colaboração de uma profissional de Design da Universidade de São Paulo (USP) uma prancha com figuras ilustrativas (Apêndice 2), para utilização na avaliação da Fala - prova de nomeação, contendo fones da língua portuguesa, preferencialmente em posição inicial na palavra. A elaboração desse material ocorreu a partir de estudo sobre a aquisição e ocorrência dos fones da língua portuguesa, por faixa etária, sendo organizado o quadro do cronograma de aquisição dos fonemas, que passou a integrar o novo protocolo.

Na etapa de validação baseada no conteúdo do teste, a maioria dos itens do novo protocolo de exame clínico foi considerada adequada, sendo obtido na 1ª. rodada, 90,5% de concordância e considerados 100% dos escores adequados, por pelo menos 90% dos especialistas (Tabela 1). Na 2ª. rodada foi apresentado aos especialistas o novo protocolo já contendo as sugestões apontadas na 1ª. rodada. Obteve-se pelo menos 70% de respostas “Concordo totalmente.

**Tabela 1:** Percentual de concordância entre os avaliadores e Índice de Validade de Conteúdo referente aos dados específicos do Protocolo de Exame Clínico Miofuncional Orofacial MMBGR

**Nº de Avaliadores que concordam na aplicação do protocolo MMBGR**	**Nº de Itens (%)**	**IVC (%)**	**p-valor**	**Nº de Escores (%)**	**IVC (%)**	**p-valor**
10	79 (57,7)	100	1,000	75 (75,0)	100	1,000
9	45 (32,8)	90	0,972	25 (25,0)	90	0,972
8	9 (6,6)	80	0,851	0 (0,0)	80	0,851
7	2 (1,5)	70	0,617	0 (0,0)	70	0,617
6	2 (1,5)	60	0,350	0 (0,0)	60	0,350

Teste Binomial Exato

**Legenda:** IVC = Índice de Validade de Conteúdo; % = frequência relativa percentual

Na etapa de validação baseada na evidência de validade baseada nos processos de resposta, evidenciou-se dificuldade na obtenção do domínio referente ao Tônus a partir do método de coleta de dados adotado na pesquisa (análise passiva das imagens editadas). Porém, de modo geral, para os demais domínios, a análise da confiabilidade das imagens mostrou concordância^(13)^ inter e intra observador, tanto de forma agrupada, como de forma mais estratificada (Tabela 2). Foram consideradas as somas dos escores atribuídos a cada item analisado do protocolo.

**Tabela 2:** Análise da Concordância inter e intra-avaliador para a aplicação do Protocolo de Exame Clínico Miofuncional Orofacial MMBGR - agrupados e estratificados por grupo etário, em meses

**Itens**	**Inter avaliador**				**Intra-avaliador**			
	**6-11**	**12-23**	**24-35**	**36-71**	**6-11**	**12-23**	**24-35**	**36-71**
	**CCI**	**CCI**	**CCI**	**CCI**	**CCI**	**CCI**	**CCI**	**CCI**
**Exame Extraoral**	0,62	0,72	0,26	0,52	0,73	0,30	0,79	0,87
FACE	0,37	0,32	0,13	0,27	0,56	-0,13	0,75	0,72
Lábios	0,66	0,81	0,59	0,80	0,74	0,33	0,83	0,56
Mandíbula	0,94	0,79	0,72	0,72	0,82	0,44	0,81	1,00
**Exame Intraoral**	0,26	0,51	0,75	0,39	0,59	0,86	0,88	0,65
Lábios	0,25	0,29	0,62	0,75	0,40	0,06	0,89	0,71
Bochecha	0,94^¥^	0,89^¥^	0,81	0,55	1,00^¥^	1,00^¥^	0,96	0,14
Língua/Fixação	0,37	0,36	0,83	0,60	0,40	0,70	0,90	0,25
Palato	0,03	0,42	0,65	0,31	0,97	0,00	0,89	0,60
Tonsilas Palatinas	1,00^£^	0,88	0,73	0,47		1,00^£^	1,00	0,94
Dentes e Oclusão	0,94^¥^	0,77	0,64	0,60	1,00^¥^	0,79	0,62	0,90
**Tônus**	0,39	0,30	0,13	0,34	0,64	0,72	0,40	0,75
**Respiração**	0,63	0,62	0,40	0,75	0,61	0,15	0,48	1,00
**Sucção/Deglutição**	0,12				0,09			
**Mastigação**		0,02	0,56	0,45		0,17	0,38	0,71
**Deglutição**	0,82	0,43	0,62	0,49	0,78	0,73	0,51	0,92
**Fala**			0,44	0,65			0,88	0,80

¥ porcentagem de concordância;

**£** número insuficiente para calcular CCI ou porcentagem de concordância

**Legenda:** CCI = Coeficiente de Correlação Intraclasse

## DISCUSSÃO

O estudo foi conduzido com o propósito apresentar o Protocolo de Exame Clínico Miofuncional Orofacial pertencente ao Protocolo MMBGR - Lactentes e Pré-escolares, incluindo sua adaptação e validação. Inicialmente foi realizada a evidência de validade baseada no conteúdo do teste que foi adaptado do Protocolo MBGR, para ser utilizado com a faixa etária entre 6 e 71 meses.

A partir da experiência da prática profissional dos autores, do referencial bibliográfico consultado, e apreciação dos especialistas, foi concluída a versão final do Protocolo.

No protocolo MMBGR Exame Clínico Miofuncional Orofacial foram excluídos itens de difícil registro na faixa etária abordada, tais como aqueles que dependiam da execução por intermédio de atender a ordem do examinador, postura corporal, medidas da face, dos movimentos mandibulares e da oclusão. Verificou-se que outro instrumento, AMIOFE^(9)^ também não realiza a aferição das medidas faciais. Por outro lado, foi acrescido o item de dentição decídua, pertinente para a população estudada, a partir do estudo da cronologia de erupção dentária^(14)^. Foram ainda acrescidos: “Utensílios utilizados na alimentação”; “Sucção”; “Deglutição de pastoso”; “Deglutição de sólido/semissólido”. Conteúdos sobre desenvolvimento da alimentação, com padrões por faixa etária e habilidades, foram fundamentados no guia alimentar para crianças brasileiras menores de 2 anos, do Ministério da Saúde do Brasil^(15)^, e em protocolos internacionais^(16,17)^.

Aspectos do padrão de amamentação e alimentação complementar, incluindo o uso de bicos artificiais e avaliação da sucção, foram fundamentados em trabalhos da própria pesquisadora^(3,18)^ e também nos manuais referência do Ministério da Saúde do Brasil^(19-21)^.

Conteúdos referentes à Comunicação e Fala, incluindo o “quadro com cronologia de ocorrência dos fones” e elaboração da “prancha de Figuras” para prova de nomeação, foram pautados nos estudos sobre o Desenvolvimento da fala, sobretudo em protocolos de avaliação de linguagem já existentes - ABFW - teste de linguagem infantil nas áreas de fonologia, vocabulário, fluência e pragmática^(22)^ e PROC: protocolo de observação comportamental: avaliação de linguagem e aspectos cognitivos infantis^(23)^. Foram enfatizados os aspectos de produção articulatória relacionada à Motricidade Orofacial, em idades precoces.

Sobre os valores de concordância obtidos na validação baseada no conteúdo do teste-do Protocolo de Exame Clínico Miofuncional Orofacial, são positivos, equiparando-se a outros estudos com instrumentos da área de Motricidade Orofacial, realizados com cálculo do IVC^(3)^.

Na etapa de validação baseada nos processos de resposta e confiabiabilidade, os valores obtidos podem também ser considerados positivos, pois a grande maioria dos itens do Exame Extraoral, Exame Intraoral, Mastigação obtiveram concordância classificada como razoável a boa, ou até mesmo Excelente. Destaca-se que os itens Respiração, Deglutição e Fala tiveram valores superiores a 0,4 em todas as faixas etárias.

Vale dizer que, para determinados domínios cuja concordância foi considerada pobre em algumas faixas etárias, os itens demonstraram concordância relativamente alta entre 60% e 90% em outras faixas de idade, contudo, dependendo da quantidade de itens, do nível de discordância intra-item e do nível de dependência entre os itens, o escore do domínio pode apresentar muitas discordâncias devido a propagação dos erros, ou seja, a soma dos erros dos itens combinados eleva muito a discordância global.

Algumas considerações precisam ser feitas a respeito da dificuldade de obtenção de concordância satisfatória entre os avaliadores para alguns itens na validação baseada na evidência de validade baseada nos processos de resposta e confiabilidade, sobretudo por se tratar de um protocolo destinado à avaliação clínica, que pode ser aplicado diretamente com o paciente, contudo, para esta pesquisa foi analisado por intermédio de imagens (estáticas e dinâmicas).

O item Tônus apresentou concordância pobre inter-avaliadores em todas as faixas etárias estudadas, evidenciando a dificuldade de validação desse aspecto a partir do método adotado (análise passiva das imagens editadas). Considera-se que a análise do Tônus mediante observação das estruturas, com respectiva mobilidade, palpação direta, além da realização das funções estomatognáticas^(24)^, é fundamental na avaliação clínica.

O item Exame Extraoral – Face também apresentou concordância pobre inter-avaliadores em todas as faixas etárias estudadas. Entretanto, a análise minuciosa dos subitens evidenciou concordância abaixo de 70% somente para alguns escores. Pondera-se sobre a dificuldade de analisar simetria e proporção facial sem utilizar os critérios antropométricos objetivos, importantes na precisão do diagnóstico na área de Motricidade Orofacial^(25)^.

Os valores de concordância pobre para Exame Intraoral em idades precoces coincidem com o fato de não se conseguir realizar o exame de orofaringe na maior parte dos lactentes, por choro e estresse. Já a avaliação da língua/fixação foi realizada com certa facilidade, mas o registro de imagem muitas vezes não evidenciou com precisão a região da inserção, e elevação da ponta da língua. Quanto à função Sucção/Deglutição, nota-se que, apesar de concordância pobre, os diversos aspectos obtiveram altos percentuais de concordância inter-avaliadores (todos acima de 74,3%), havendo apenas dificuldade na classificação do estado comportamental do lactente no início da mamada.

Na faixa etária de 12 a 23 meses a principal dificuldade quanto aos valores de concordância pobre entre os avaliadores foi em Mastigação, referentes à observação do Padrão mastigatório - unilateral/bilateral; ao Escape de alimento; e às Contrações Musculares não esperadas. Pondera-se que o padrão de mastigação infantil, inerente à fisiologia normal do desenvolvimento pode ter gerado controvérsias na análise. A introdução de alimentos na consistência sólida nessa faixa etária é importante, mas existe um processo gradual de modificação na aceitação da alimentação, sendo que entre os doze e os vinte e quatro meses há a exploração de diferentes texturas e sabores^(26)^. Assim, apesar da mastigação já poder ser avaliada em lactentes entre 12 e 23 meses, o Protocolo Exame Clínico Miofuncional Orofacial do MMBGR obteve boa confiabilidade quanto à função Mastigação apenas a partir dos 24 meses, ou seja, para pré-escolares.

Na faixa etária de 24 a 35 meses, as principais dificuldades quanto aos valores de concordância pobre entre os avaliadores foram apenas nos itens Face e Tônus, conforme ocorreu para outras faixas etárias. Na faixa etária de 36 a 71 meses a principal dificuldade foi em Exame Intraoral: Palato, o que pode ter ocorrido pela análise de uma única imagem destinada a esse item. Pondera-se que a análise a partir do exame direto com o paciente, seja feita considerando-se a observação e compreensão de outros aspectos, tais como condições de oclusão dentária, observação da postura de língua e modo respiratório. Considera-se um item fundamental de ser mantido no Protocolo MMBGR.

O Protocolo MMBGR - Lactentes e Pré-escolares brasileiros foi elaborado seguindo padrões de desenvolvimento da população falante do português brasileiro. O uso para outra população necessita de validação transcultural. Novos estudos voltados às próximas etapas de validação que ainda não foram realizadas são importantes, incluindo a validação de critério e constructo do novo instrumento aqui apresentado.

Por fim, acredita-se que este instrumento apresentado preenche uma importante lacuna para a clínica da Motricidade Orofacial e suas pesquisas, ampliando o conhecimento científico na Fonoaudiologia.

## CONCLUSÃO

No presente artigo foi apresentada a adaptação e validação do Exame Clínico Miofuncional Orofacial, que compõe o Protocolo MMBGR – Lactentes e Pré-Escolares, possibilitando a utilização do novo instrumento para faixa etária de 06 a 71 meses de vida, que ainda não estava contemplada quanto a protocolos específicos em MO.

O protocolo Exame Clínico Miofuncional Orofacial, que integra o protocolo MMBGR – Lactentes e Pré-escolares, demonstrou ter validade no conteúdo do teste, nos processos de resposta e confiabilidade para lactentes e pré-escolares sem queixa de distúrbios miofuncionais, para a maioria dos itens analisados.

## Apêndice 1 PROTOCOLO MMBGR – Lactentes e Pré-Escolares: *EXAME CLÍNICO*

PROTOCOLO MMBGR

EXAME MIOFUNCIONAL OROFACIAL COM ESCORES

LACTENTES E PRÉ-ESCOLARES (6 meses até 5 anos e 11 meses)

Andréa Monteiro Correia Medeiros, Irene Queiroz Marchesan, Katia Flores Genaro, Giédre Berretin-Felix

1. IDENTIFICAÇÃO

**Table:**

Nome: ______________________________________________________________		N°: __________________
Data do Exame: _____ / _____ / _____	Idade: ____anos e ___ meses	DN: _____ / _____ / _____
Peso corporal: ________kg	Altura corporal: _______ m	IMC: ___ (peso [kg]/estatura [m]^2^)
Responsável: ____________________	Nome da mãe/pai:___________________________________________________	

**2. EXAME EXTRAORAL** [ ] Soma dos pontos da face, lábios e mandíbula *(melhor resultado = 0 e pior = 20*)

**Face** [ ] Soma dos pontos (*melhor resultado = 0 e pior = 10*) Análise facial subjetiva em norma frontal

**Table:**

	**Simétrico**	**Assimétrico**	***Descrever***
**Plano infraorbitário**	(0)	(1)	
**Região zigomática**	(0)	(1)	
**Asas do nariz**	(0)	(1)	
**Bochechas**	(0)	(1)	
**Sulco nasolabial**	(0)	(1)	
**Lábio superior**	(0)	(1)	
**Comissura dos lábios**	(0)	(1)	
**Lábio inferior**	(0)	(1)	
**Mento**	(0)	(1)	
**Mandíbula (corpo e ramo)**	(0)	(1)	

**Lábios** [ ] Soma dos pontos (*melhor resultado = 0 e pior = 9*)

**Table:**

**Postura habitual:**		(0) fechados		(1) fechados com tensão	(2) ora abertos, ora fechados
		(2) entreabertos		(2) fechados em contato dentário	(3) abertos
**Forma:**	▪ Superior:	(0) normal (1º arco do cupido)		(1) em asa de gaivota (1º e 2º arcos do cupido)	
	▪ Inferior:	(0) normal		(1) com eversão leve	(2) com eversão acentuada
**Mucosa externa:**		(0) normal	(1) com saliva	(1) ressecada	(2) ferida

**Mandíbula** [ ] Soma dos pontos (*melhor resultado = 0 e pior = 1*)

**Table:**

**No repouso:**	(0) elevada	(1) abaixada

Observação *(Exame Extraoral*): _____________________________________________________________________________________

**3. EXAME INTRAORAL** [ ] Soma dos pontos de lábios, bochechas, língua, palato, tonsilas palatinas, dentes e oclusão

(*melhor resultado = 0 e pior = 42*): *até 23 meses de idade*

(*melhor resultado = 0 e pior = 56*): *a partir de 24 meses de idade*

**Lábios** [ ] Soma dos pontos (*melhor resultado = 0 e pior = 5*)

**Table:**

**Mucosa interna:**	(0) normal		(1) com marcas dentárias		(2) ferida
**Frênulo superior:**	▪ Fixação no rebordo alveolar:			(0) adequada	(1) baixa
	▪ Espessura:	(0) adequada		(1) alterada: ___________________________________________________	

Observação: __________________________________________________________________________________________________

**Bochechas** [ ] Soma dos pontos (*melhor resultado = 0 e pior = 6): até 23 meses de idade*

(*melhor resultado = 0 e pior = 8): a partir de 24 meses de idade*

**Table:**

❒ *Avaliar em lactentes (até 23 meses de idade)*				
**Mucosa:**	(0) normal	(1) monilíase oral (“sapinho”) D	(2) ferida D	
		(1) monilíase oral (“sapinho”) E	(2) ferida E	
❒ *Avaliar em pré-escolares (a partir de 24 meses de idade)*				
**Mucosa:**	(0) normal	(1) marcas dentárias/aparelho D	(1) linha alba D	(2) ferida D
		(1) marcas dentárias/aparelho E	(1) linha alba E	(2) ferida E

Observação: __________________________________________________________________________________________________

**Língua** [ ] Soma dos pontos (*melhor resultado = 0 e pior = 13): até 23 meses de idade*

(*melhor resultado = 0 e pior = 16): a partir de 24 meses de idade*

**Table:**

❒ *Avaliar em lactentes (até 23 meses de idade)*						
**Postura habitual:**	❒ não visível			(1) comprimida na cavidade oral		
	(0) contida na cavidade oral			(1) interposta entre dentes e/ou rebordos gengivais		
**Mucosa:**	(0) normal		(1) geográfica	(1) com fissuras	(2) ferida (região):________________________	
❒ *Avaliar em pré-escolares (a partir de 24 meses de idade)*						
**Postura habitual:**	❒ não visível			(1) no assoalho	(1) ponta baixa e dorso alto	(2) interdental
**Mucosa:**	(0) normal	(1) geográfica		(1) com fissuras	(2) ferida (região): ________________________	
	(1) marcada por dentes (região): ___________________				(1) marcada por aparelho (região): ___________	

Observação: __________________________________________________________________________________________________

Frênulo

**Table:**

**Fixação**	▪ no assoalho, visível a partir:				(0) das carúnculas			(1) da crista alveolar	
	▪ na língua:	(0) no terço médio				(1) entre o terço médio e o ápice		(2) no ápice	
**Forma do ápice ao elevar língua:**				(0) arredondada			(1) quadrada ou retangular		(2) formato de coração
							(1) ligeira fenda no ápice		(3) não se eleva
**Outras características:**			(0) não há			(1) submucoso ou posterior		(2) espesso	

Observação: __________________________________________________________________________________________________

**Palato** [ ] Soma dos pontos (*melhor resultado = 0 e pior = 10)*

**Table:**

**Duro:**		▪ Profundidade:			(0) adequada		(1) reduzida (baixo)		(2) aumentada (alto)	
		▪ Largura:			(0) adequada		(1) aumentada (larga)		(2) reduzida (estreitada)	
**Véu palatino:**			▪ Simetria:			(0) presente	(1) ausente (descrever): ______________________________________________			
			▪ Extensão:			(0) adequada	(1) longa		(2) curta	
**Úvula:**	▪ Aspecto:			(0) adequada			(1) longa	(1) hipoplásica	(1) sulcada	(2) bífida

Observação: __________________________________________________________________________________________________

**Tonsilas palatinas** [ ] Soma dos pontos (*melhor resultado = 0 e pior = 4*)

**Table:**

**Presença:**	❒ presentes	❒ removidas	❒ não visíveis
**Tamanho:**	(0) adequado	(1) hipertrofia D	(1) hipertrofia E
**Coloração:**	(0) adequada	(1) hiperemia D	(1) hiperemia E

Observação: __________________________________________________________________________________________________

**Dentes e Oclusão** [ ] Soma dos pontos (*melhor resultado = 0 e pior = 4): até 23 meses de idade*

(*melhor resultado = 0 e pior = 13): a partir de 24 meses de idade*

**Table:**

**Dentes:**	▪ Superior: direito ____						esquerdo ____		▪ Inferior: direito____		esquerdo: ____	
**Saúde oral:**	▪ Dentes:				(0) boa			(1) regular	(2) ruim			
	▪ Gengivas:				(0) boa			(1) regular	(2) ruim			
❒ *Avaliar em pré-escolares (a partir de 24 meses de idade)* **Avaliar esse item/subitem somente quando houver a dentição decídua completa, com presença dos segundos molares.*												
**Linha média:**			(0) adequada					(1) desviada D	(1) desviada E			
**Relação transversal*:**			(0) adequada					(1) mordida cruzada posterior D		(1) mordida cruzada posterior E		
**Relação horizontal:**			(0) adequada					(1) sobressaliência	(1) mordida cruzada anterior			(1) mordida de topo
**Relação vertical:**			(0) adequada					(1) sobremordida	(1) mordida aberta posterior D*			
			(1) mordida de topo					(1) mordida aberta anterior	(1) mordida aberta posterior E*			
**Relação entre caninos*:**				(0) classe I D				(1) classe II D	(1) classe III D			
				(0) classe I E				(1) classe II E	(1) classe III E			
**Uso de aparelho:**		❒ não				❒ sim: Tipo:__________________________________________________________________						

Observação: __________________________________________________________________________________________________

**4. TÔNUS** [ ] Soma dos pontos *(melhor resultado = 0 e pior = 6)* (*realizar observação visual e palpação*)

**Table:**

	**Normal**	**Diminuído**	**Aumentado**
**Lábio superior:**	(0)	(1)	(1)
**Lábio inferior:**	(0)	(1)	(1)
**Mento:**	(0)	(1)	(1)
**Língua:**	(0)	(1)	(1)
**Bochecha D:**	(0)	(1)	(1)
**Bochecha E:**	(0)	(1)	(1)

Observação: __________________________________________________________________________________________________

**5. FUNÇÕES OROFACIAIS** [ ] Soma dos pontos da respiração, sucção, mastigação, deglutição e fala

**Respiração** [ ] Soma dos pontos *(melhor resultado = 0 e pior = 2)*

Se alterada, está relaciona à: [ ] hábito [ ] possível fator obstrutivo [ ] outra: ____________________

**Table:**

**Modo:**	(0) nasal	(1) oronasal	(2) oral
**Fluxo nasal** *(Usar o espelho)*:		❒ semelhante entre as narinas	❒ assimetria: [ ] leve [ ] moderada [ ] acentuada

Observação: __________________________________________________________________________________________________

**Sucção/Deglutição** [ ] soma dos pontos *(melhor resultado = 0 e pior = 22)*

**Table:**

❒ *Avaliar até 23 meses de idade, em lactentes que ainda estejam em amamentação (peito) ou que utilizem mamadeira*																	
**Via de oferta:**		[ ] peito				[ ] mamadeira (descrever o tipo de bico: ____________________________________________											
**Líquido utilizado:**		❒ água				❒ leite	❒ suco		❒ outro: _________________________________________								
**Estado comportamental** (início):						(0) alerta	(1) sono leve/sonolento				(1) agitado/irritado					(2) choro	
**Padrão de sucção:**			(0) presente - grupos regulares					(1) presente - grupos irregulares						(2) sucção esporádica			(3) ausente
**Força de sucção:**			(0) forte			(1) média	(2) fraca		(3) ausente								
**Postura dos lábios:**			(0) vedamento total				(1) vedamento parcial					(2) sem vedamento					
**Contração do orbicular:**				(0) adequada			(1) pouca		(1) acentuada				(2) ausente				
**Contração do mentual:**				(0) ausente			(1) pouca		(1) acentuada								
**Movimento da língua:**				❒ não observável			(0) organizado		(1) desorganizado: _________________________________								
**Movimento de cabeça:**				(0) ausente			(1) presente										
**Contenção do líquido:**				(0) adequada			(1) inadequada, com pouco escape								(2) inadequada, com muito escape		
**Ritmo:**	(0) satisfatório			(1) rápido (sem pausas)			(1) lento		(2) ausente								
**Ruído:**	(0) ausente			(1) presente													
**Coordenação: sucção/respiração/deglutição:**							(0) adequada			(1) engasgo				(1) tosse			
**Resíduos após deglutir:**					(0) ausente		(1) presente										

Observação: __________________________________________________________________________________________________

**Mastigação** [ ] soma dos pontos *(melhor resultado = 0 e pior = 13)*

Se alterada, a origem é: [ ] funcional [ ] estrutural [ ] outra: _______________________________

**Mastigação de sólido** [ ] *(alimento contendo pedaços maiores, na mesma consistência da dieta da família)*

**Mastigação de semissólido** [ ] *(alimento contendo pedaços cortados bem pequenos e macios ou desfiados)*

(*se for o padrão esperado ou o item não se aplicar para a idade, considerar zero)*

❒ *Avaliar a partir de 12 meses de idade*

**Table:**

**Alimento utilizado:**				❒ pão			❒ biscoito (tipo):_____________			❒ fruta em pedaço			❒ comida da família/escola	
							❒ Outro: __________________________________________________________________							
**Incisão:**		(0) anterior					(1) lateral		(2) não realiza			(1) outra: ____________________________		
**Trituração:**			(0) dentes posteriores						(0) dentes anteriores na ausência de molares					(0) eficiente
			(1) amassamento com a língua						(1) dentes anteriores na presença de molares					(2) ineficiente
**Padrão mastigatório:**					(0) unilateral/bilateral alternado				(1) bilateral simultâneo					
					(0) unilateral preferencial				(2) unilateral crônico					
**Fechamento labial:**					(0) sistemático				(1) assistemático					
**Mastigação ruidosa:**					(0) não		(1) sim							
**Escape do alimento:**					(0) não		(1) sim							
**Contrações musculares não esperadas:**								(0) ausentes			(1) presentes (descrever): _______________________			
**Reflexos orais exacerbados:**						(0) ausentes			(1) presente (gag)			(1) presente (mordida)		
**Ritmo:**	(0) adequado					(1) lento		(1) rápido						

Observação: ______________________________________________________________________________________________

**Deglutição** [ ] soma dos pontos líquido + pastoso ( *melhor resultado = 0 e pior = 37)*

soma dos pontos líquido+ sólido + semissólido *(melhor resultado = 0 e pior = 32)*

Se alterada, a origem é: [ ] funcional [ ] estrutural [ ] outra: _______________________________

(**se for o padrão esperado para a idade, considerar zero. Observação válida para todas as consistências)*

**Deglutição de sólido** [ ] *(alimento contendo pedaços maiores, na mesma consistência da dieta da família)*

**Deglutição de semissólido** [ ] *(alimento contendo pedaços cortados bem pequenos e macio sou desfiados)*

soma dos pontos *(melhor resultado = 0 e pior = 17)*

**Table:**

❒ *Avaliar a partir de 12 meses de idade: Conforme aceitação de dieta. Quando já aceita sólido, não avaliar semissólido*									
**Alimento utilizado:**				❒ pão		❒ biscoito (tipo): __________	❒ fruta em pedaço	❒ comida da família/escola	
				❒ Outro: _________________________________________________________________________________					
**Via de oferta:**				❒ mãos		❒ colher	❒ garfo	❒ outra: _________________	
**Prontidão:**		(0) presente (abre a boca alimento se aproxima/toca os lábios)						(1) ausente	
**Postura dos lábios:**				(0) fechados		(1) lábio inferior em contato com dentes superiores		(2) abertos	
						(1) parcialmente fechados			
**Postura da língua*:**				❒ não observável		(0) atrás dos dentes	(1) contra os dentes	(2) entre os dentes	
**Movimento da língua*:**					❒ não observável	(0) anteroposterior	(1) amassamento	(1) posteroanterior	(2) ausente
**Contenção do alimento:**					(0) adequada	(1) parcial	(2) inadequada – com escape		
**Contração do orbicular:**					(0) adequada	(1) pouca	(2) acentuada		
**Contração do mentual:**					(0) ausente	(1) pouca	(2) acentuada		
**Movimento de cabeça:**					(0) ausente	(1) presente			
**Ritmo*:**	(0) uma deglutição					(1) duas deglutições	(2) deglutições múltiplas		
**Ruído:**	(0) ausente				(1) presente				
**Coordenação:**			(0) adequada			(1) engasgo	(1) tosse		
**Resíduos após deglutir:**					(0) ausente	(1) presente			

Observação: __________________________________________________________________________________________________

**Deglutição de pastoso** *(papa/ purê, alimento amassado)* [ ] soma dos pontos *(melhor resultado = 0 e pior = 22)*

**Table:**

❒ *Avaliar até 11 meses de idade: (Poderá avaliar até 23 meses, em lactentes que se alimentem na consistência pastosa)*																			
**Alimento utilizado:**				❒ papa			❒ purê		❒ alimento amassado (qual): _________________________________________										
**Via de oferta:**				❒ colher			❒ outra: __________________________________________________________________												
**Prontidão:**		(0) presente *(abre a boca quando a colher se aproxima/toca os lábios)*												(1) ausente					
**Reflexo de mordida:**				(0) presente					(1) exacerbado					(1) ausente					
**Reflexo de gag:**				(0) presente					(1) exacerbado					(1) ausente					
**Postura dos lábios:**				(0) fechados					(1) lábio inferior em contato com dentes superiores									(2) abertos	
									(1) fechados parcialmente										
**Movimento dos lábios:**					(0) adequado *(move lábio superior para remover alimento da colher)*									(1) pouco (exagerado)			(1) exagerado		
**Postura da língua*:**				❒ não observável					(0) atrás dos dentes				(1) contra os dentes			(2) entre os dentes*****			
**Movimento da língua*:**					❒ não observável				(0) anteroposterior		(1) amassamento*****				(1) posteroanterior				(2) ausente
**Volume do alimento:**					(0) satisfatório				(1) aumentado		(1) diminuído								
**Contenção do alimento:**						(0) adequada			(1) inadequada – com escape										
**Contração do orbicular:**						(0) adequada			(1) pouca		(2) acentuada								
**Contração do mentual:**						(0) ausente			(1) pouca		(2) acentuada								
**Movimento de cabeça:**						(0) ausente			(1) presente										
**Ritmo:**	(0) uma deglutição								(1) duas deglutições		(2) deglutições múltiplas								
**Ruído:**	(0) ausente								(1) presente										
**Coordenação:**			(0) adequada					(1) engasgo		(1) tosse									
**Resíduos após deglutir:**						(0) ausente			(1) pouco			(2) muito							

Observação: __________________________________________________________________________________________________

**Deglutição de pastoso** *(não utilizar mamadeira para avaliar)* [ ] soma dos pontos *(melhor resultado = 0 e pior = 15)*

**Table:**

❒ *Avaliar a partir de 12 meses de idade*									
**Líquido utilizado:**				❒ água			❒ leite	❒ suco	❒ outro: ___________________
**Via de oferta:**		❒ copo comum					❒ copo com tampa	❒ copo com válvula	❒ outra: ___________________
**Postura dos lábios:**					(0) fechados		(1) lábio inferior em contato com dentes superiores		(2) abertos
							(1) fechados parcialmente		
**Postura da língua*:**					❒ não observável		(0) atrás dos dentes	(1) contra os dentes	(2) entre os dentes*****
**Volume do líquido:**					(0) satisfatório		(1) aumentado	(1) diminuído	
**Contenção do líquido:**						(0) adequada	(1) inadequada – com escape		
**Contração do orbicular:**						(0) adequada	(1) pouca	(2) acentuada	
**Contração do mentual:**						(0) ausente	(1) pouca	(2) acentuada	
**Movimento de cabeça:**						(0) ausente	(1) presente		
**Ritmo:**	(0) sequencial						(1) gole por gole		
**Ruído:**	(0) ausente						(1) presente		
**Coordenação:**			(0) adequada				(1) engasgo	(1) tosse	(1) alteração vocal/voz molhada

Observação: __________________________________________________________________________________________________

**Fala** [ ] Soma dos pontos - produção de fones/fonemas +aspectos gerais da fonoarticulação *(melhor resultado = 0 e pior = 21)*

Característica: [ ] Fonológica [ ] Fonética/Fonológica [ ] Fonética

Se alteração fonética, a origem é: [ ] funcional [ ] estrutural [ ] neuromuscular [ ] outra: _______________

**Produção de fones/fonemas** [ ] Soma dos pontos *(melhor resultado = 0 e pior = 6)*

**Table:**

❒ *Avaliar a partir de 12 meses de idade:* (0) ausente (1) presente (*se esperado para a idade, considerar zero)*			
**Nomeação de figuras/Repetição** [ ] Somar todos os pontos *(melhor resultado = 0 e pior = 3)* *Usar Protocolo MMBGR - Figuras para Nomeação* () substituição () omissão () distorção			
**Fala semidirigida** [ ] Somar todos os pontos *(melhor resultado = 0 e pior = 3)* *Dizer o nome e idade/ Falar sobre a escola ou uma brincadeira/ Contar sobre uma viagem ou passeio* () substituição () omissão () distorção			
Fones/Fonemas e características: preencher o quadro abaixo			
**No caso de substituição de ponto articulatório:**		[ ] auditivamente perceptível	[ ] visualmente perceptível
**No caso de distorção, relaciona-se à:**	[ ] ausência/pouca vibração do ápice da língua		[ ] elevação do dorso
	[ ] vibração múltipla do ápice da língua		[ ] rebaixamento do dorso
	[ ] língua interdental: () anterior () lateral		

Observação: __________________________________________________________________________________________________

**Prova terapêutica** *Solicitar a repetição de sílabas que contenham os sons alterados, combinados com a vogal “e”*

Observar se ocorre modificação na emissão quando é fornecido o modelo correto

**Table:**

**Fone testado**	**a produção não se altera**	**a produção melhora**	**a produção torna-se adequada**
	[ ]	[ ]	[ ]
	[ ]	[ ]	[ ]
	[ ]	[ ]	[ ]

Observação: __________________________________________________________________________________________________

**Aspectos gerais da fonoarticulação** [ ] Soma dos pontos *(melhor resultado = 0 e pior = 15)*

**Table:**

❒ *Avaliar a partir de 36 meses de idade:*													
**Saliva:**		(0) deglutida					(1) acumulada na comissura direita e/ou esquerda				(2) espirra		(3) baba
							(1) acumulada no lábio inferior						
**Abertura da boca:**						(0) adequada			(1) reduzida		(1) aumentada		
**Posição da língua na fala:**							(0) adequada		(1) no assoalho		(2) posteriorizada		
									(2) interdental (projeção)		(2) ápice baixo e elevadas laterais		
**Movimento da mandíbula:**							(0) adequado		(1) desvio à direita		(1) desvio à esquerda		(1) anteriorização
**Movimento dos lábios:**							(0) adequado		(1) reduzido		(1) exagerado		
**Movimento da língua:**							(0) adequado		(1) reduzido				
**Velocidade:**				(0) adequada					(1) aumentada		(1) reduzida		
**Ressonância:**				(0) equilíbrio oronasal					(1) uso reduzido nasal: () leve		() moderado	() grave	
				(1) laringofaríngea					(1) uso excessivo nasal: () leve		() moderado	() grave	
**Coordenação pneumofonoarticulatória:**									(0) adequada		(1) alterada ________________________________		
**Articulação:**				(0) precisa					(1) imprecisão assistemática		(2) imprecisão sistemática		
No caso de imprecisão, relaciona-se à:													
[ ]tônus [ ]audição			[ ]velocidade de fala [ ]respiração oronasal					[ ]quantidade de saliva [ ]má oclusão		[ ]fadiga muscular [ ]redução da abertura da boca		[ ]alteração neurológica [ ]outro:_______________________	
**Voz**	*▪ Pitch:*				[ ]Adequado			[ ]Grave		[ ]Agudo			
	*▪ Loudness:*				[ ]Adequado			[ ]Forte		[ ]Fraco			
	*▪* Tipo:				[ ]Adequado			[ ]Alterado					

Observação: __________________________________________________________________________________________________

_____________________________________________________________________________________________________________

**Table:**

**Roteiro para registo de imagens**						
**Imagens Estáticas**						
- Face:	[ ] Visão frontal sem correção da postura da cabeça				[ ] Visão frontal com correção da postura da cabeça	
- Lábios:	[ ] Em repouso – habitual		[ ] Mucosa interna		[ ] Frênulo labial superior	
- Bochechas:	[ ] Mucosa interna direita		[ ] Mucosa interna esquerda			
- Língua:	[ ] Exteriorizada *(fora da cavidade oral)*					
	[ ] Frênulo *(língua elevada sem tocar o palato)*				[ ] Frênulo *(língua elevada com manobra)*	
- Palato:	[ ] Duro					
- Dentes:	[ ] Arcada Superior		[ ]Arcada Inferior			
- Oclusão:	[ ] Anterior		[ ] Lado Direito		[ ] Lado Esquerdo	
- Outras:	[ ] À critério do examinador					
**Imagens Dinâmicas**						
- Sucção:	[ ] Amamentação (peito)			[ ] Mamadeira		
- Mastigação:	[ ] Boca aberta após mastigar e antes de deglutir					
- Deglutição:	[ ] Líquido	[ ] Pastoso		[ ] Sólido/Semissólido		[ ] Boca aberta após deglutir *(resíduo)*
- Fala:	[ ] Semidirigida	[ ] Nomeação de figuras/repetição				
- Orofaringe:	[ ] Palato mole	[ ] Úvula		[ ] Tonsilas Palatinas		

**Table:**

**Dados coletados de exames: _________________________________________________________________**				
__________________________________________________________________________________________				
__________________________________________________________________________________________				
__________________________________________________________________________________________				
__________________________________________________________________________________________				
__________________________________________________________________________________________				
**Exames solicitados (justificativa): ____________________________________________________________**				
__________________________________________________________________________________________				
__________________________________________________________________________________________				
__________________________________________________________________________________________				
__________________________________________________________________________________________				
__________________________________________________________________________________________				
**Diagnóstico fonoaudiológico: ________________________________________________________________**				
__________________________________________________________________________________________				
__________________________________________________________________________________________				
__________________________________________________________________________________________				
__________________________________________________________________________________________				
__________________________________________________________________________________________				
__________________________________________________________________________________________				
**Prognóstico:**	[ ] favorável	[ ] limitado	[ ] desfavorável
__________________________________________________________________________________________				
__________________________________________________________________________________________				
__________________________________________________________________________________________				
**Encaminhamento para outros profissionais (área e justificativa): __________________________________**				
__________________________________________________________________________________________				
__________________________________________________________________________________________				
__________________________________________________________________________________________				
__________________________________________________________________________________________				
**Plano terapêutico: __________________________________________________________________________**				
__________________________________________________________________________________________				
__________________________________________________________________________________________				
__________________________________________________________________________________________				
__________________________________________________________________________________________				
__________________________________________________________________________________________				
__________________________________________________________________________________________				
__________________________________________________________________________________________				

Resumo do Exame Miofuncional Orofacial - MMBGR - Lactentes e Pré-escolares

Andréa Monteiro Correia Medeiros, Irene Queiroz Marchesan, Katia Flores Genaro, Giédre Berretin-Felix

**Table:**

**EXAME EXTRAORAL - Faixa etária (meses/ano)**	**06-11**	**12-23**	**24-35**	**36-71**
		**(1 ano)**	**(2 anos)**	**(3-5 anos)**
**(melhor resultado = 0 e pior = 20)**	**[ ]**	**[ ]**	**[ ]**	**[ ]**
	**0-20**	**0-20**	**0-20**	**0-20**
Face (*melhor resultado = 0 e pior = 10)*	[ ]	[ ]	[ ]	[ ]
Lábios (*melhor resultado = 0 e pior = 9)*	[ ]	[ ]	[ ]	[ ]
Mandíbula (*melhor resultado = 0 e pior =1)*	[ ]	[ ]	[ ]	[ ]
**EXAME INTRAORAL**	**[ ]**	**[ ]**	**[ ]**	**[ ]**
**(melhor resultado = 0 e pior = 42/56)**	**0-42**	**0-42**	**0-56**	**0-56**
Lábios *(melhor resultado = 0 e pior = 5)*	[ ]	[ ]	[ ]	[ ]
Bochechas *(melhor resultado = 0 e pior = 6/8)*	[ ]	[ ]	[ ]	[ ]
Língua *(melhor resultado = 0 e pior = 13/16)*	[ ]	[ ]	[ ]	[ ]
Palato *(melhor resultado = 0 e pior = 10)*	[ ]	[ ]	[ ]	[ ]
Tonsilas palatinas *(melhor resultado = 0 e pior = 4)*	[ ]	[ ]	[ ]	[ ]
Dentes e Oclusão *(melhor resultado = 0 e pior = 4/13)*	[ ]	[ ]	[ ]	[ ]
**TÔNUS**	**[ ]**	**[ ]**	**[ ]**	**[ ]**
**(melhor resultado = 0 e pior = 6)**	**0-6**	**0-6**	**0-6**	**0-6**
Lábios *(superior+inferior) (melhor resultado = 0 e pior = 2)*	[ ]	[ ]	[ ]	[ ]
Mento *(melhor resultado = 0 e pior = 1)*	[ ]	[ ]	[ ]	[ ]
Língua *(melhor resultado = 0 e pior = 1)*	[ ]	[ ]	[ ]	[ ]
Bochechas *(direita+esquerda) (melhor resultado = 0 e pior = 2)*	[ ]	[ ]	[ ]	[ ]
**FUNÇÕES OROFACIAIS**	**[ ]**	**[ ]**	**[ ]**	**[ ]**
**(melhor resultado = 0 e pior =46/92/53/68)**	**0-46**	**0-92**	**0-53**	**0-68**
Respiração *(melhor resultado = 0 e pior = 2)*	[ ]	[ ]	[ ]	[ ]
Sucção/Deglutição *(melhor resultado = 0 e pior = 22)*	[ ]	[ ]	___	___
Mastigação *(melhor resultado = 0 e pior = 13)*	___	[ ]	[ ]	[ ]
Deglutição Líquido + pastoso (*melhor resultado = 0 e pior = 37)*				
Deglutição Líquido + sólido/semissólido *(melhor resultado = 0 e pior = 32)*				
Deglutição semissólido/sólido *(melhor resultado = 0 e pior = 17)*	___	[ ]	[ ]	[ ]
Deglutição pastoso *(melhor resultado = 0 e pior = 22)*	[ ]	[ ]	___	___
Deglutição líquido *(melhor resultado = 0 e pior = 15)*	___	[ ]	[ ]	[ ]
Fala (melhor resultado = 0 e pior = 6/21)				
Produção de fones/fonemas *(melhor resultado = 0 e pior = 6)*	___	___	[ ]	[ ]
Aspectos gerais da fonoarticulação *(melhor resultado = 0 e pior = 15)*	___	___	___	[ ]
**ESCORE TOTAL**	**[ ]**	**[ ]**	**[ ]**	**[ ]**

Fonoaudiólogo: ____________________________________ CRFª: ___________

## Apêndice 2 Prancha (Frente e Verso) – Figuras para Nomeação – Protocolo MMBGR – Lactentes e Pré-Escolares

Andréa Monteiro Correia Medeiros, Irene Queiroz Marchesan, Katia Flores Genaro, Giédre Berretin-Felix
