# Supplementary material for: ERRATUM: MMBGR Protocol - infants and preschoolers: myofunctional orofacial clinic examination
Source: Codas. 2023 Jan 6;35(1):e20220190. doi: 10.1590/2317-1782/20212022190en (PMC10010420; doi:10.1590/2317-1782/20212022190en)
Supplement: Translation

# Translation: ERRATA: Protocolo MMBGR – lactentes e pré-escolares: exame clínico miofuncional orofacial

**DOI:** 10.1590/2317-1782/20212022190pt

Devido ao erro honesto dos autores o artigo “Protocolo MMBRG – lactentes e pré-escolares: exame clínico miofuncional orofacial” (DOI https://doi.org/10.1590/2317-1782/20212020325), publicado em CoDAS 2022;34(5):e20200325, foi publicado com erros.

**No título em português, onde se lê:**

Protocolo MMBRG – lactentes e pré-escolares: exame clínico miofuncional orofacial

**Leia-se:**

Protocolo MMBGR – lactentes e pré-escolares: exame clínico miofuncional orofacial

**No título em inglês, onde se lê:**

MMBRG Protocol – infants and preschoolers: myofunctional orofacial clinic examination

**Leia-se:**

MMBGR Protocol – infants and preschoolers: myofunctional orofacial clinic examination

**Na versão em inglês, nas páginas 3, 4, 10, 11, 13 e 15, onde se lê:**

Pasty

**Leia-se:**

Pudding

**Na versão em português, página 11, no primeiro quadro “Deglutição de pastoso” , onde se lê:**

**Movimento dos lábios:** (0) adequado (*move lábio superior para remover alimento da colher*) (1) pouco (exagerado) (1) exagerado

**Leia-se:**

**Movimento dos lábios:** (0) adequado (*move lábio superior para remover alimento da colher*) (1) pouco (1) exagerado

**Na versão em português, página 11, no segundo quadro, onde se lê:**

**Deglutição de pastoso** (não utilizar mamadeira para avaliar)

**Leia-se:**

**Deglutição de líquido** (não utilizar mamadeira para avaliar)

Os autores pedem desculpas pelos erros.
